# Blood pressure lowering and risk of new-onset type 2 diabetes: an individual participant data meta-analysis

**DOI:** 10.1016/S0140-6736(21)01920-6

**Published:** 2021-11-13

**Authors:** Milad Nazarzadeh, Zeinab Bidel, Dexter Canoy, Emma Copland, Malgorzata Wamil, Jeannette Majert, Karl Smith Byrne, Johan Sundström, Koon Teo, Barry R Davis, John Chalmers, Carl J Pepine, Abbas Dehghan, Derrick A Bennett, George Davey Smith, Kazem Rahimi, Blood Pressure Lowering Treatment Trialists’ Collaboration

**Affiliations:** a Deep Medicine, Oxford Martin School, University of Oxford, Oxford, UK; b Nuffield Department of Women's and Reproductive Health, Medical Science Division, University of Oxford, Oxford, UK; c Clinical Trial Service Unit and Epidemiological Studies Unit, Nuffield Department of Population Health, University of Oxford, Oxford, UK; d NIHR Oxford Biomedical Research Centre, Oxford University Hospitals NHS Foundation Trust, Oxford, UK; e International Agency for Research on Cancer/WHO, Genomic Epidemiology Branch, Lyon, France; f Clinical Epidemiology Unit, Department of Medical Sciences, Uppsala University, Uppsala, Sweden; g Population Health Research Institute, McMaster University, Hamilton, ON, Canada; h The University of Texas School of Public Health, Houston, TX, USA; i The George Institute for Global Health, University of New South Wales, Sydney, NSW, Australia; j Department of Medicine, University of Florida, Gainesville, FL, USA; k Department of Biostatistics and Epidemiology, School of Public Health, Imperial College London, London, UK; l MRC Integrative Epidemiology Unit, University of Bristol, Bristol, UK

## Abstract

**Background:**

Blood pressure lowering is an established strategy for preventing microvascular and macrovascular complications of diabetes, but its role in the prevention of diabetes itself is unclear. We aimed to examine this question using individual participant data from major randomised controlled trials.

**Methods:**

We performed a one-stage individual participant data meta-analysis, in which data were pooled to investigate the effect of blood pressure lowering per se on the risk of new-onset type 2 diabetes. An individual participant data network meta-analysis was used to investigate the differential effects of five major classes of antihypertensive drugs on the risk of new-onset type 2 diabetes. Overall, data from 22 studies conducted between 1973 and 2008, were obtained by the Blood Pressure Lowering Treatment Trialists’ Collaboration (Oxford University, Oxford, UK). We included all primary and secondary prevention trials that used a specific class or classes of antihypertensive drugs versus placebo or other classes of blood pressure lowering medications that had at least 1000 persons-years of follow-up in each randomly allocated arm. Participants with a known diagnosis of diabetes at baseline and trials conducted in patients with prevalent diabetes were excluded. For the one-stage individual participant data meta-analysis we used stratified Cox proportional hazards model and for the individual participant data network meta-analysis we used logistic regression models to calculate the relative risk (RR) for drug class comparisons.

**Findings:**

145 939 participants (88 500 [60·6%] men and 57 429 [39·4%] women) from 19 randomised controlled trials were included in the one-stage individual participant data meta-analysis. 22 trials were included in the individual participant data network meta-analysis. After a median follow-up of 4·5 years (IQR 2·0), 9883 participants were diagnosed with new-onset type 2 diabetes. Systolic blood pressure reduction by 5 mm Hg reduced the risk of type 2 diabetes across all trials by 11% (hazard ratio 0·89 [95% CI 0·84–0·95]). Investigation of the effects of five major classes of antihypertensive drugs showed that in comparison to placebo, angiotensin-converting enzyme inhibitors (RR 0·84 [95% 0·76–0·93]) and angiotensin II receptor blockers (RR 0·84 [0·76–0·92]) reduced the risk of new-onset type 2 diabetes; however, the use of β blockers (RR 1·48 [1·27–1·72]) and thiazide diuretics (RR 1·20 [1·07–1·35]) increased this risk, and no material effect was found for calcium channel blockers (RR 1·02 [0·92–1·13]).

**Interpretation:**

Blood pressure lowering is an effective strategy for the prevention of new-onset type 2 diabetes. Established pharmacological interventions, however, have qualitatively and quantitively different effects on diabetes, likely due to their differing off-target effects, with angiotensin-converting enzyme inhibitors and angiotensin II receptor blockers having the most favourable outcomes. This evidence supports the indication for selected classes of antihypertensive drugs for the prevention of diabetes, which could further refine the selection of drug choice according to an individual's clinical risk of diabetes.

**Funding:**

British Heart Foundation, National Institute for Health Research, and Oxford Martin School.

## Introduction

Diabetes affects about 9% of the adult population worldwide, with a rising prevalence in many regions.^1^ Patients with diabetes often have elevated blood pressure, and a disproportionately high risk of developing cardiovascular disease.^2^, ^3^ Although blood pressure lowering is an established strategy for preventing microvascular and macrovascular events in people with type 2 diabetes,^4^ its benefit for the prevention of diabetes itself has been less clear. Thus, whether elevated blood pressure is a modifiable risk factor for diabetes remains to be established.

Combined evidence from cohort studies suggests that each 20 mm Hg higher systolic blood pressure is associated with a 77% increased risk of type 2 diabetes.^5^ However, the causality of that association remains uncertain, as observational evidence is prone to confounding and reverse causation. Evidence from randomised controlled trials^6^, ^7^, ^8^ and mendelian randomisation investigations^9^ has been unclear as well, with previous studies having insufficient statistical power and not considering potentially opposing effects of different blood pressure lowering drug classes on the risk of type 2 diabetes. For instance, individual studies have shown that the renin–angiotensin–aldosterone system inhibitors might decrease the risk of new-onset type 2 diabetes,^10^, ^11^, ^12^, ^13^ whereas diuretics could increase that risk (appendix p 13).^14^, ^15^ Consequently, it remains uncertain whether the protective or adverse events associated with blood pressure lowering medications are due to blood pressure reduction or off-target effects of the drugs. This uncertainty is also reflected in clinical guidelines that do not provide clear recommendations for pharmacological or non-pharmacological blood pressure reduction as a strategy for the prevention of type 2 diabetes.^16^, ^17^, ^18^

**Research in context**

**Evidence before this study**

PubMed, Web of Science, and Scopus were searched for published data related to “hypertension”, “blood pressure”, and “diabetes”**,** with no language restrictions between Jan 1, 1966, and Sept 1, 2021. We found no meta-analysis of randomised trials that reported the effect of blood pressure reduction itself on incident diabetes. Several observational cohort studies were identified but with conflicting findings. For the question of drug-class specific effects, we found several individual trial reports, including a network meta-analysis. However, it remains uncertain whether the protective or adverse effects on diabetes risk are due to blood pressure reduction per se or off-target effects of each of the drug classes.

**Added value of this study**

We used large-scale individual participant data from randomised controlled trials to investigate the effect of blood pressure lowering and the differential effects of five major classes of antihypertensives on risk of new-onset type 2 diabetes. A fixed level of 5 mm Hg reduction in systolic blood pressure reduced the risk of diabetes by 11%. This treatment effect constituted quantitatively and qualitatively diverging effects of major antihypertensive drug classes. In analysis of specific drug classes versus placebo, angiotensin-converting enzyme inhibitors and angiotensin II receptor blockers had the strongest protective effect on the risk of diabetes. For calcium channel blockers no material effect was found, while β blockers and thiazide diuretics increased the risk.

**Implications of all the available evidence**

This study suggests that blood pressure lowering can help prevent diabetes in addition to its well established beneficial effects in reducing cardiovascular events. The relative magnitude of reduction per 5 mm Hg systolic blood pressure lowering was similar to those reported for prevention of major cardiovascular events, which will strengthen the case for blood pressure reduction through lifestyle interventions known to reduce blood pressure, and blood pressure lowering treatments with drugs, and possibly device therapies. The differing effects of some drug classes also support decision making for drug choice according to an individual's risk profile. In particular, angiotensin-converting enzyme inhibitors and angiotensin II receptor blockers should become the drugs of choice when clinical risk of diabetes is of concern, whereas β blockers and thiazide diuretics should be avoided where possible. This study also encourages further research into identification and clinical testing of alternative mechanisms for diabetes prevention that are not necessarily targeting hyperglycaemia. Thus, this research could provide additional avenues for curbing the growing burden of diabetes.

We used individual-level data from large-scale randomised trials of blood pressure lowering treatments to assess the effect of blood pressure lowering on the risk of new-onset type 2 diabetes and to establish the comparative effects of five major blood pressure lowering drug classes on that risk.

## Methods

### Overview

In this individual participant data meta-analysis, we used the resources of the Blood Pressure Lowering Treatment Trialists’ Collaboration (BPLTTC), a collaboration of principal investigators and triallists of major randomised controlled trials of pharmacological blood pressure lowering treatment.^19^, ^20^ For this study, we included all primary and secondary prevention trials that used a specific class or classes of antihypertensive drugs versus placebo or other classes of blood pressure lowering medications that had at least 1000 persons-years of follow-up in each randomly allocated arm (appendix p 32). All participants with a known diagnosis of diabetes at baseline or trials conducted in patients with prevalent diabetes were excluded. New-onset type 2 diabetes was defined based on the diagnostic criteria reported by each trial (appendix p 15). Participants were grouped into the intervention and comparator treatment arms. For placebo-controlled trials, the placebo arm was considered as the comparator and the active arm as the intervention. For head-to-head trials that compared two or more drug classes, the arm with the greater systolic blood pressure reduction was considered as the intervention and the other as the comparator. The summary characteristics of the included trials are shown in the appendix (p 16).

The BPLTTC obtained approval to conduct this collaborative research from the Oxford Tropical Research Ethics Committee (reference 545-14). In addition, as part of the complementary genetic analysis, we used data resources from the UK Biobank that obtained informed consent from the study participants and approval from its institutional review board.

### Statistical analysis

A one-stage individual participant data meta-analysis framework was used for statistical analysis.^21^ We used stratified Cox proportional hazard models, with fixed treatment effects and participants as the unit of analysis.^22^ We standardised the effect sizes for a 5 mm Hg reduction in systolic blood pressure between randomised groups as a convenient round value close to the weighted mean of systolic blood pressure reduction across all trials.^19^, ^21^ Standardisation of effect size is useful when the aim is to assess the effects of blood pressure reduction through pooling of the data from different trials with differing amounts of blood pressure reduction.^21^ Patients entered the analysis at the date of randomisation and were followed up until the earliest occurrence of type 2 diabetes, death, study exit, or end of the trial. Kaplan-Meier survival curves were used to compare the probability of survival during the follow-up time. A subgroup analysis was done to assess the heterogeneity of effect by body-mass index categories. Likelihood-ratio test was used to test heterogeneity of treatment effect across subgroups of body-mass index categories at baseline. We used funnel plot and Egger's regression test to check whether inability to obtain data from all trials might lead to acquisition bias. The risk of bias for each trial was assessed by the revised Cochrane risk-of-bias tool and has been reported in a previous study.^21^

We did several sensitivity and supplementary analyses to check the robustness of findings. We conducted stratified analysis by different diabetes ascertainment methods reported by each trial to assess consistency of findings across different ascertainment methods. We additionally reported a one-stage Cox proportional hazards model, which included random effects terms and adjusted for multiple levels of potential confounders. The absolute risk reductions were calculated using a Poisson regression model with identity link to show treatment effects on an absolute scale. Finally, in a complementary analysis, we re-assessed the effect of blood pressure lowering through mendelian randomisation, as an independent framework that uses naturally randomised genetic variants to mimic blood pressure lowering treatment effect (appendix pp 5–7).^23^

The presence of diverse blood pressure lowering drug classes with different mechanisms of action limits the usefulness of conventional multiple pairwise comparisons (comparing one class of drug versus other classes) for clinical decision making. Therefore, to estimate the effect of each class of drug, we fitted a Bayesian fixed-effect network meta-analysis model to compare the effect of different classes of antihypertensive drugs with placebo on the risk of type 2 diabetes.^24^, ^25^ The network meta-analysis method combines all direct (within-trial comparisons) and indirect evidence (between-trial comparisons) and makes it possible to compare the efficacy of different treatments with a common comparator such as placebo; an approach that is particularly useful when direct evidence is scarce for this comparison from individual trials. For this analysis, we did not standardise the analyses for the magnitude of blood pressure reduction in each trial to account for the total off-target (or non-blood pressure mediated) effects and blood pressure mediated effects of the different drug classes. Therefore, the estimated effects from these sets of analyses provide a summary of all mechanisms that drugs might have, related and unrelated to blood pressure lowering. We estimated the effect of the five major blood pressure lowering drug classes, including angiotensin-converting enzyme inhibitors (ACEIs), angiotensin II receptor blockers (ARBs), β blockers, calcium channel blockers (CCBs), and thiazide diuretics. We used a logistic regression model to estimate the relative risk (RR) for each possible comparison using individual-level information for each trial. To run the network meta-analysis model, we used the Markov chain Monte-Carlo simulation approach with four chains and 100 000 iterations after an initial burn-in of 10 000.^26^ In a complementary analysis, a mendelian randomisation approach was used to replicate the effect of each class of drug through genetic variants in druggable genes (appendix p 10).

The prespecified analysis plan was approved by the BPLTTC steering committee and collaborators before releasing the data for analysis. Statistical analyses were performed using R, version 4.0.2.

### Role of the funding source

The funders of the study had no role in study design, data collection, data analysis, data interpretation, or writing of the report.

## Results

**Figure 1:**
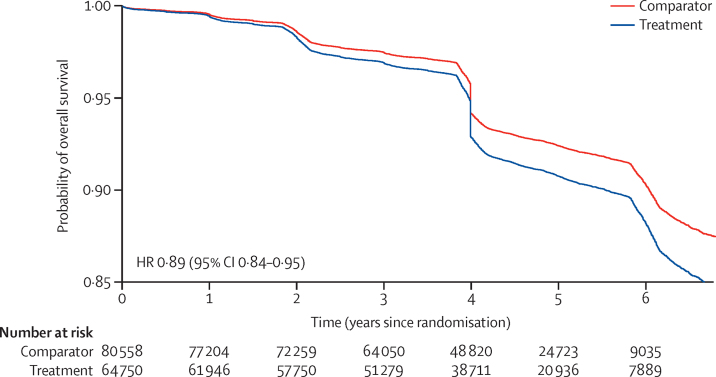
Kaplan-Meier estimates of survival in the intervention and comparator groups The curve has been truncated at 6 years after randomisation and adjusted for the systolic blood pressure reduction reached at the trial level. All participants with a known diagnosis of diabetes were excluded at baseline. HR=hazard ratio.

**Figure 2:**
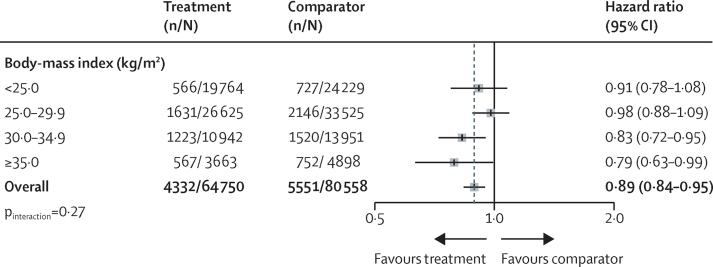
Blood pressure lowering treatment and risk of new-onset type 2 diabetes, by body-mass index categories at baseline The forest plot shows the hazard ratios and 95% CIs per 5 mm Hg reduction in systolic blood pressure.

The characteristics of participants included in the individual participant data meta-analysis are shown in the table. 145 939 (88 500 [60·6%] men and 57 429 [39·4%] women) randomly assigned participants from 19 trials were included in the one-stage individual participant data meta-analysis. 22 trials were included in the individual participant data network meta-analysis (appendix p 16). For survival analysis, we excluded 631 participants with missing information for follow-up time. Over a median follow-up of 4·5 years (IQR 2·0), we identified 9883 cases of new-onset type 2 diabetes. The incidence rate for developing a new-onset type 2 diabetes event per 1000 person-years was 16·44 (95% CI 16·01–16·87) in the comparator group and 15·94 (15·47–16·42) in the intervention group. The hazard ratio and 95% CI for diagnosis of new-onset type 2 diabetes during follow-up for a 5 mm Hg reduction in systolic blood pressure was 0·89 (95% CI 0·84–0·95; figure 1, appendix p 34), equating to an 11% reduction in risk for type 2 diabetes. We did not find any meaningful heterogeneity of treatment effects by body-mass index in the subgroup analysis (figure 2).

**Table:** Baseline characteristics of participants included in the one-stage individual participant data meta-analysis

		**Comparator group (n=80 887)**	**Treatment group (n=65 042)**
Sex			
	Women	31 788 (39·3%)	25 641 (39·4%)
	Men	49 099 (60·7%)	39 401 (60·6%)
Age, years		65·5 (9·7)	64·9 (9·9)
Systolic blood pressure, mm Hg		153 (22·1)	154 (21·8)
Diastolic blood pressure, mm Hg		89 (12·4)	89 (12·5)
Categories of systolic blood pressure, mm Hg			
	<120	3827/80 855 (4·7%)	2826/65 019 (4·3%)
	120–129	6724/80 855 (8·3%)	5195/65 019 (8·0%)
	130–139	10 250/80 855 (12·7%)	8019/65 019 (12·3%)
	140–149	15 408/80 855 (19·1%)	11 925/65 019 (18·3%)
	150–159	14 224/80 855 (17·6%)	11 040/65 019 (17·0%)
	160–169	12 688/80 855 (15·7%)	11 153/65 019 (17·2%)
	≥170	17 734/80 855 (21·9%)	14 861/65 019 (22·9%)
Body-mass index, kg/m^2^			
	<18·5	888/76 520 (1·2%)	692/60 933 (1·1%)
	18·5–24·9	23 303/76 520 (30·5%)	19 048/60 933 (31·3%)
	25·0–29·9	33 480/76 520 (43·8%)	26 588/60 933 (43·6%)
	≥30	18 849/76 520 (24·6%)	14 605/60 933 (24·0%)
Comorbidity			
	Peripheral vascular disease	888/21 107 (4·2%)	882/20 295 (4·3%)
	Atrial fibrillation	4915/80 890 (6·1%)	4616/65 049 (7·1%)
	Chronic kidney disease	5919/29 626 (20·0%)	5581/29 154 (19·1%)
	Cerebrovascular disease	15 794/63 482 (24·9%)	14 383/55 322 (26·0%)
	Ischaemic heart disease	22 791/80 889 (28·2%)	17 012/65 048 (26·2%)
Previous use of non-study medications			
	Angiotensin-converting enzyme inhibitor	12 479/30 968 (40·3%)	9507/24 623 (38·6%)
	Angiotensin II receptor blocker	1695/18 344 (9·2%)	1640/17 451 (9·4%)
	Calcium channel blocker	11 877/37 136 (32·0%)	9563/30 716 (31·1%)
	Diuretic	7800/37 138 (21·0%)	6529/30 716 (21·3%)
	β blocker	14 590/37 135 (39·3%)	11 251/30 716 (36·6%)
	α blocker	1110/30 541 (3·6%)	917/24 206 (3·8%)
	Antiplatelet drug	14 264/21 060 (67·7%)	9611/14 690 (65·4%)
	Anticoagulant	2902/29 166 (9·9%)	2564/22 834 (11·2%)
	Lipid-lowering treatment	14 189/34 305 (41·4%)	10 310/29 748 (34·7%)
Median follow-up duration, years		4·5 (1·9)	4·5 (2·0)

Data are n (%), mean (SD), or median (IQR).

There was no evidence of acquisition bias (appendix p 35). Stratified analysis by different diabetes ascertainment methods provided evidence against non-differential outcome ascertainment between the randomised treatment groups (appendix p 28). There was no material change after adjustment for baseline characteristics or after accounting for random effects terms in the Cox model (appendix p 30). The findings based on the absolute risk scale were consistent with the reported relative effects (appendix p 36).

**Figure 3:**
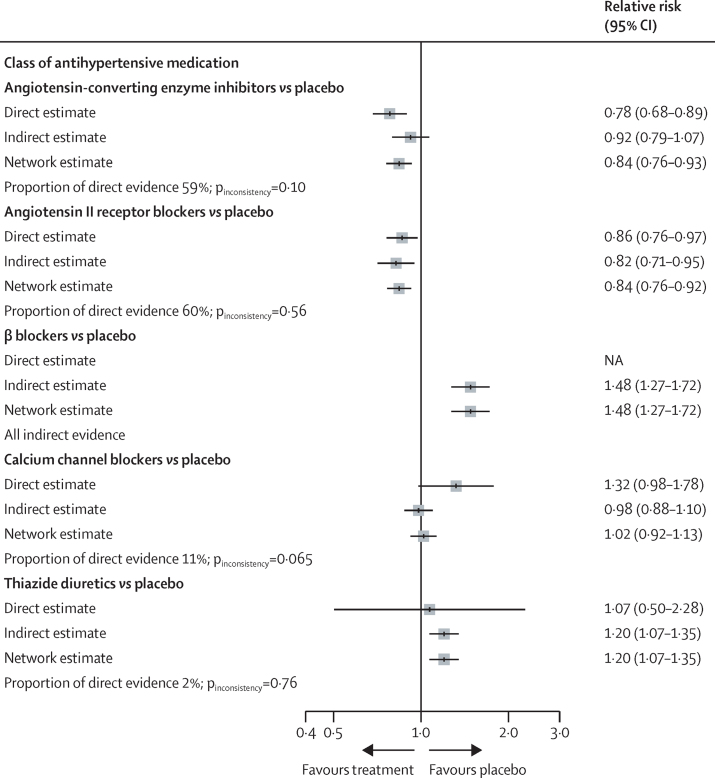
Estimated effect of major antihypertensive drug classes on the risk of new-onset type 2 diabetes The effect sizes did not standardise for blood pressure reduction between trials to account for off-target effects or non-blood pressure mediated effects of the different drug classes.

22 trials that evaluated antihypertensive drug treatment effects and collected diagnostic information for incident type 2 diabetes were included in this analysis (appendix p 16). Of the 22 included trials, eight were placebo-controlled, and 14 were head-to-head drug class comparison trials. The calculated effect sizes for each trial and structure of the dataset used for Bayesian network meta-analysis are shown in the appendix (p 31)**.** We found that ACEIs and ARBs reduced the risk of type 2 diabetes compared with placebo, with a RR of 0·84 (95% CI 0·76–0·93, 59% direct evidence) for ACEIs and RR 0·84 (0·76–0·92, 60% direct evidence) for ARBs. The network estimates showed no effect for CCBs compared with placebo (RR 1·02 [95% CI 0·92–1·13], 11% direct evidence), whereas β blockers (RR 1·48 [1·27–1·72], 0% direct evidence) and thiazide diuretics (RR 1·20 [1·07–1·35], 2% direct evidence) were found to increase the risk of type 2 diabetes compared with placebo (figure 3).

In complementary analysis using mendelian randomisation, consistent with evidence from randomised controlled trials, each 5 mm Hg genetically influenced lower systolic blood pressure was associated with a 12% lower risk of type 2 diabetes (RR 0·88 [95% CI 0·84–0·92]; appendix pp 37–45]). Additionally, the results of mendelian randomisation were in line with the network meta-analysis of randomised controlled trials, in which we found a decrease in the risk with ACEIs and ARBs, null effect with CCBs, and increased risk with β blockers (appendix p 46). The genetic evidence for thiazide diuretics did not provide adequate statistical power for replication (appendix p 46).)

## Discussion

In this large-scale analysis of individual participant data from randomised clinical trials, we found evidence for the preventive effect of blood pressure lowering on the risk of type 2 diabetes, with an 11% reduction in the risk of new-onset type 2 diabetes per 5 mm Hg lower systolic blood pressure. When investigating the effects of major antihypertensive drug classes, we found that in comparison to placebo, ACEIs and ARBs reduced the risk of type 2 diabetes, β blockers and thiazide diuretics increased the risk of the disease, and CCBs had no material influence on type 2 diabetes risk. Findings from randomised clinical trials were largely confirmed in independent complementary analysis using genetic data.

Previous observational evidence has shown conflicting associations between elevated blood pressure and risk of new-onset type 2 diabetes. In a prospective cohort analysis of 7735 participants with 12·8 years of follow-up, no association was found between elevated systolic blood pressure and type 2 diabetes.^27^ Similarly, the Whitehall II study, a prospective occupational cohort study that included 10 308 participants at baseline, showed no increased risk of type 2 diabetes per unit increase of systolic blood pressure.^28^ By contrast, a meta-analysis of cohort studies that included about 4·7 million participants reported a 77% greater risk of type 2 diabetes per 20 mm Hg higher systolic blood pressure.^5^ However, the observational nature of these findings precluded drawing firm conclusions about causality. Similarly, evidence from previous mendelian randomisation studies investigating the effect of genetically determined higher systolic blood pressure on type 2 diabetes has been contradictory, likely due to low statistical power (appendix p 13).^9^, ^29^, ^30^ Previous reports from randomised controlled trials have not been able to resolve this issue, in part because analyses were focused on drug classes as opposed to blood pressure reduction.^8^, ^15^ These uncertainties have led to the absence of clear recommendations from international guideline committees on the adoption of blood pressure lowering via pharmacological or non-pharmacological interventions for the prevention of type 2 diabetes.^16^, ^17^, ^18^

Our study fills this gap in evidence using individual participant data from randomised controlled trials and assessing effects for a standardised fixed degree of blood pressure reduction. With consistent results from both randomised controlled trials and genetic analyses, we have shown that elevated blood pressure is indeed a modifiable risk factor for new-onset type 2 diabetes in people without a diagnosis of diabetes, with a relative effect size similar to those seen for the prevention of major cardiovascular disease.^21^, ^31^, ^32^ The evidence that blood pressure reduction is linked to diabetes presents clinicians and health policy makers with an opportunity to modify disease risk, for instance, either through the use of appropriate antihypertensive medications or by promoting lifestyle behaviours known to reduce blood pressure such as by maintaining a healthy weight through physical activity and a balanced diet.

These findings have important implications also in the context of the generally disappointing pharmacological interventions through glucose-modifying pathways and the observed increase in risk of type 2 diabetes with lipid-lowering treatments as another major strategy for prevention of cardiovascular disease.^21^, ^31^, ^33^ There is evidence from randomised controlled trials^34^, ^35^ that lipid-lowering treatment, particularly statin therapy, increases the risk of new-onset type 2 diabetes by 10%.^34^, ^35^ This effect, which has also been confirmed in genetic analyses,^36^, ^37^ is considered as one of the main side-effects of lipid-lowering. In this context, the finding that blood pressure lowering is typically expected to reduce the risk of type 2 diabetes will add to the importance of this strategy in at-risk populations.

Different antihypertensive drugs might affect the risk of type 2 diabetes through blood pressure lowering as well as their class-specific effects through other off-target mechanisms. Thus, when investigating their effect, it is prudent to consider their overall effect irrespective of the degree of blood pressure reduction in trials. By design, non-randomised comparisons and individual randomised controlled trials are not well suited for comparing the effect of drug classes, as non-randomised comparisons might be subject to bias and individual randomised controlled trials often have insufficient statistical power and typically investigate the effect of a single drug. A previous network meta-analysis used summary data from randomised controlled trials to explore this question. It reported a preventive effect associated with ARBs compared with placebo (odds ratio [OR] 0·75 [95% CI 0·61–0·91]), and an excess risk associated with diuretics compared with placebo (OR 1·30 [1·07–1·58]). No clear effect was found for ACEIs (OR 0·87 [0·75–1·01]), CCBs (OR 0·97 [0·82–1·15]), or β blockers (OR 1·17 [0·98–1·40]) compared with placebo.^15^

Our individual participant data study extends earlier findings by providing more precise estimates of effect sizes, which led to some qualitatively different results. More specifically, we found strong evidence for the effect of ACEIs and ARBs on reducing the risk of new-onset type 2 diabetes, suggesting that renin–angiotensin–aldosterone system deactivation could causally lower the risk of the disease. Consistent with the previous report,^15^ we found evidence for the absence of an effect of CCBs on type 2 diabetes risk. Finally, the evidence from our network analysis showed that in comparison to placebo, β blockers and thiazide diuretics increase the risk of new-onset type 2 diabetes. This adverse diabetes effect supports recommendations to classify these agents as low priority for treating hypertension when the risk of diabetes or pre-diabetes is of clinical concern.^16^, ^17^ Furthermore, we validated these findings independently through mendelian randomisation analysis, with the exception of thiazide diuretics in which the number of known genes, which mimic the effect of this drug (appendix p 10) was relatively small and, hence, the randomised controlled trial results remain the best source of evidence. This triangulation adds further weight to the robustness and importance of our individual participant data meta-analysis.^38^

Although the exact biological pathways through which elevated blood pressure causes new-onset type 2 diabetes are unknown, several potential mechanisms have been reported. Among others, insulin resistance, vascular inflammation, and endothelial dysfunction, which tend to precede the clinical manifestation of diabetes^39^, ^40^, ^41^ are all pathophysiological consequences of hypertension.^42^, ^43^ For instance, insulin resistance might play a central role in the cross-talk between metabolism and cardiovascular pathways.^44^ Other pathways, such as increased activation of the sympathetic nervous system and chronic inflammation leading to endothelial dysfunction, have also been suggested as links between hypertension and the risk of diabetes.^45^ Notably, the effect of antihypertensive drug classes on these mediating factors is variable and might explain their differential off-target effects. As an example, renin angiotensin inhibition has been shown to reduce the concentration of inflammatory markers, independently of the blood pressure lowering effect, which might enhance their protective effect on diabetes.^42^, ^46^ Other plausible biological mechanisms for their protective effect is the improvement of insulin resistance through the suppression of reactive oxygen species.^47^ For β blockers and thiazide diuretics, although there is no certainty about the biological pathway for diabetes risk, studies have suggested that modification of insulin secretion and carbohydrate metabolism in β blockers,^48^, ^49^ and potassium depletion in thiazide diuretics^50^ could play a role. Likewise, CCBs have either no known material effects on these mediating mechanisms or might have additional pathophysiological sequelae that negate their blood pressure lowering effect.^51^ Further experimental studies are required to explore these and other possible mechanisms. In addition, by showing that the risk of diabetes can be modified with drugs that are not targeting hyperglycaemia, this study encourages future research to identify additional molecular targets for the prevention of diabetes.

This study has some limitations. First, we did not assess the effect of combinations of drugs with opposing or synergistic effects on type 2 diabetes risk because of the limited information available. However, we believe that understanding the effect of a single class of drug is still of major clinical importance, even for selecting the most appropriate combinations of treatment. Relatedly, information on dosages and post-randomisation treatment were incomplete, and hence, the results reflect the effects of drug dosages across the duration of the studies. We were unable to obtain data from several eligible randomised controlled trials, but we found no evidence of data acquisition bias in our findings. Another limitation is the case ascertainment as diabetes was not the primary endpoint in the included trials. However, randomised trials are robust to bias from case ascertainment and the main risk resulting from incomplete case identification is the dilution of the true treatment effects.^52^ To further assess this issue, we extracted information on the method of diabetes ascertainment at baseline, diabetes ascertainment during follow-up, and the calculated incidence rate. We found that the overall incidence rate of diabetes was lower in trials that relied largely on adverse event reports than those with more complete laboratory testing. However, relative risk reductions were similar across trial groups with differing methods of case ascertainment; a finding that further supports the validity of overall estimation and the study conclusions.

Using randomised evidence from major pharmacological blood pressure lowering trials, this study has shown consistent evidence to suggest that the preventive effect of blood pressure reduction on type 2 diabetes risk is causal, and therefore reducing blood pressure is likely to prevent new-onset type 2 diabetes. This evidence also supports the indication for selected classes of antihypertensive drugs for the prevention of type 2 diabetes, which could further refine the selection of drug choice according to an individual's risk profile. In particular, ACEIs and ARBs should be considered as having the most favourable outcomes when clinical risk of diabetes is a concern.

## Data sharing

The governance of the BPLTTC has been reported previously. The BPLTTC is governed by the University of Oxford's policies on research integrity and codes of practice and follows the university's policy on the management of research data and records. Scientific activities based on the BPLTTC dataset are overseen by the BPLTTC steering committee. All data shared with the BPLTTC will be considered confidential and will not be provided to any third party. Requests for data should be made directly to the data custodians of individual trials. Information about individual projects is posted on the BPLTTC website. All bona fide researchers can apply to use the UK Biobank dataset for health-related research. A guide to access is also provided on the UK Biobank website. Access to summary statistics for diabetes is available from cnsgenomics and for the International Consortium for Blood Pressure from the database of Genotypes and Phenotypes.

## Declaration of interests

KR reports personal fees from the journals *Heart* and *PLoS Medicine,* outside the submitted work. JS reports stock ownership in companies providing services to Itrim, Amgen, Janssen, Novo Nordisk, Eli Lilly, Boehringer Ingelheim, Bayer, Pfizer, and AstraZeneca, outside the submitted work. JC reports a grant from the National Health and Medical Research Council of Australia, outside the submitted work. All other authors declare no competing interests.
