# Supplementary material for: Blood pressure lowering and risk of new-onset type 2 diabetes: an individual participant data meta-analysis
Source: Lancet. 2021 Nov 13;398(10313):1803–10. doi: 10.1016/S0140-6736(21)01920-6 (PMC8585669; doi:10.1016/S0140-6736(21)01920-6)
Supplement: Supplementary appendix [file mmc1.pdf]

THE LANCET

Supplementary appendix

This appendix formed part of the original submission and has been peer reviewed. We post it as supplied by the authors.

Supplement to: Nazarzadeh M, Bidel Z, Canoy D, et al. Blood pressure lowering and risk of new-onset type 2 diabetes: an individual participant data meta-analysis. *Lancet* 2021; **398:** 1803–10.

Supplementary materials

Blood pressure lowering and risk of new-onset type 2 diabetes: an individual participant data meta-analysis

Milad Nazarzadeh [1,2], Zeinab Bidel [1,2], Dexter Canoy [1,2,3], Emma Copland [1,2], Malgorzata Wamil [1], Jeannette Majert [1,2], Karl Smith Byrne [4], Johan Sundström [5], Koon Teo [6], Barry R Davis [7], John Chalmers [8], Carl J. Pepine [9], Abbas Dehghan [10], Derrick A Bennett [3,11], George Davey Smith [12], Kazem Rahimi [1,2,3] *, The Blood Pressure Lowering Treatment Trialists' Collaboration †

[1] Deep Medicine, Oxford Martin School, University of Oxford, UK
[2] Nuffield Department of Women's and Reproductive Health, Medical Science Division, University of Oxford, UK
[3] NIHR Oxford Biomedical Research Centre, Oxford University Hospitals NHS Foundation Trust, Oxford, UK
[4] International Agency for Research on Cancer/World Health Organization (IARC/WHO), Genomic Epidemiology branch, Lyon, France
[5] Clinical Epidemiology Unit, Department of Medical Sciences, Uppsala University, Sweden
[6] Population Health Research Institute, McMaster University, Hamilton, Ontario, Canada
[7] The University of Texas School of Public Health, Houston, Texas, USA
[8] The George Institute for Global Health, University of New South Wales, Sydney, Australia
[9] Department of Medicine, University of Florida, Gainesville, Florida, USA
[10] Department of Biostatistics and Epidemiology, School of Public Health, Imperial College London, UK
[11] Clinical Trial Service Unit and Epidemiological Studies Unit, Nuffield Department of Population Health, University of Oxford, Oxford, UK
[12] MRC Integrative Epidemiology Unit, University of Bristol, Bristol, UK

*** Corresponding author:** Kazem Rahimi

Deep Medicine, Nuffield Department of Women's and Reproductive Health, University of Oxford, Oxford, United Kingdom; email address: kazem.rahimi@wrh.ox.ac.uk; Tel: +44 (0) 1865 617200; Fax: +44 (0) 1865 617202; Postal code and address: 1st Floor, Hayes House, 75 George Street, Oxford, OX1 2BQ, United Kingdom

† The Blood Pressure Lowering Treatment Trialists' Collaboration

Steering Committee: Kazem Rahimi (Chair), Koon Teo, Barry R Davis, John Chalmers, Carl J Pepine

Collaborating Trialists: Ajay K Gupta, Alberto Zanchetti, Ale Algra, Amanda Adler, Anushka Patel, Barry R Davis, Bertram Pitt, Carl J Pepine, Christopher Reid, Christopher J Bulpitt, Christopher R Palmer, Edmund Lewis, Eivind Berge, Folkert W Asselbergs, Frank P J Brouwers, Gianpaolo Reboldi, Giuseppe Mancia, Giuseppe Remuzzi, Henry Black, Hiromi Rakugi, Hiromichi Suzuki, Hiroshi Ogawa, Jamie Dwyer, Jan Lanke, Jan A Staessen, Jeffrey Cutler, Joachim Schrader, John Kostis, John Chalmers, Julia B Lewis, Kenji Ueshima, Kim Fox, Kizuku Kuramoto, Kristian Wachtell, Larry Agodoa, Lars H Lindholm, Lindon Wing, Lutgarde Thijs, Marc Allan Pfeffer, Maria H Mehlum, Mark Woodward, Masao Ishii, Masunori Matsuzaki, Michel Lievre, Morris Brown, Neil R Poulter, Nigel S Beckett, Paolo Verdecchia, Paul Helton, Peter Sever, Peter Sleight, Piero Ruggenenti, Ray Estacio, Richard B Devereaux, Robert Fagard, Robert Schrier, Robert P Byington, Rury R Holman, Salim Yusuf, Seiji Umemoto, Stephan Lueders, Stephen MacMahon, Steven Nissen, Stevo Julius, Sverre E Kjeldsen, Takao Saruta, Takayoshi Ohkubo, Toshio Ogihara, Tsuguya Fukui, Wiek H van Gilst, William C Cushman, Yoshihiko Kanno, Yoshiki Yui, Yutaka Imai, Zhen-Yu Zhang

Other members: Craig Anderson, Colin Baigent, Barry Morton Brenner, Rory Collins, Dick de Zeeuw, Jacobus Lubsen, Ettore Malacco, Bruce Neal, Vlado Perkovic, Anthony Rodgers, Peter Rothwell, Gholamreza Salimi-Khorshidi, Johan Sundström, Fiona Turnbull, Giancarlo Viberti, Jiguang Wang

Contents

Method S1. Mendelian randomisation of blood pressure lowering per se on risk of diabetes 5

Method S2. Details of the International Consortium for Blood Pressure genome-wide association study 6

Method S3. Details of statistical methods and sensitivity analyses for Mendelian randomisation study 7

Method S4. The genetic approaches used to replicate the effect of each antihypertensive drug class using genetic analysis. 10

Table S1. Selected published reports on the effect of blood pressure lowering per se and specific drug class effect on risk of new-onset type 2 diabetes. 13

Table S2. Diagnostic criteria for the definition of type 2 diabetes in each trial. 15

Table S3. General characteristics of trials included in the individual patient data meta-analysis as well as Bayesian network meta-analysis. 16

Table S4. Genetic variants selected as an instrumental variable for systolic blood pressure 18

Table S5. Post-hoc sensitivity analysis of the effect of systolic blood pressure lowering per se on the risk of new-onset type 2 diabetes, stratified by different diabetes ascertainment methods reported by each trial. 28

Table S6. Post-hoc one-stage Cox proportional hazards model included random effects terms and adjusted for multiple potential confounders. 30

Table S7. Structure of data used for Bayesian network meta-analysis. 31

Figure S1. Trial selection flowchart. 32

Figure S2. The genetic variant selection workflow for systolic blood pressure. 33

Figure S3. Forest plot showing the effect of systolic blood pressure lowering per se on the risk of new-onset type 2 diabetes, overall and separately for each trial. 34

Figure S4. Funnel plot for assessment of publication (acquisition) bias on the effect of blood pressure reduction and risk of new-onset type 2 diabetes. 35

Figure S5. Absolute risk reduction for the effect of each major antihypertensive drug class on the risk of new-onset type 2 diabetes. 36

Figure S6. Effect of systolic blood pressure lowering per se on the risk of new-onset type 2 diabete. 37

Figure S7. Mendelian randomisation estimates for the association between genetically predicted 5-mmHg systolic blood pressure reduction and diabetes as the main outcome, and coronary heart disease, myocardial infarction, and ischemic stroke as positive control outcomes. 38

Figure S8. Scatter plot of genetic variant-outcome associations versus variant-exposure associations for the association between systolic blood pressure and risk of type 2 diabetes. 39

Figure S9. Funnel plot of variants, showing each variant causal estimate against instrument strength. 40

Figure S10. The association between systolic blood pressure and risk of diabetes estimated by random-effect inverse variance weighted and applied various sensitivity analysis methods of two-sample Mendelian randomisation, before (main analysis) and after excluding outlier variants (sensitivity analysis). 41

Figure S11. Leave-one-out plot to assess if a single variant is driving the association between systolic blood pressure and diabetes.......... 42

Figure S12. Multivariable Mendelian randomisation results unadjusted and adjusted for the anthropometric measures to check the possibility of collider bias in association between systolic blood pressure and diabetes. 43

Figure S13. Sensitivity analysis to assess the impact of using overlap dataset for the discovery of candidate instrumental variable for exposure. 44

Figure S14. The association between systolic blood pressure and risk of diabetes replicated by one-sample Mendelian randomisation.......... 45

Figure S15. Association of genetically influenced systolic blood pressure reduction overall and for each major class of antihypertensive medications, with type 2 diabetes as the main outcome, and coronary heart disease as the positive control. 46

References 48

Method S1. Mendelian randomisation of blood pressure lowering per se on risk of diabetes.

For this complementary analysis, the exposure was genetically-influenced systolic blood pressure used as an instrumental variable which was estimated using genetic variants with minor allele frequency >0.01 that were independently (linkage disequilibrium $r^2 < 0.05$) associated with systolic blood pressure at a genome-wide significance level ($P < 5\times10^{-8}$). Overall, 246 genetic variants were selected from a genome-wide association studies (GWAS) meta-analysis, including over one million participants of European ancestry (**Table S4 and Figure S2**).[1] Because of the overlap between the GWAS selected for exposure and outcome (UK Biobank contributing to both),[1,2] and to avoid weak instrument bias,[3] we extracted the corresponding beta coefficients and standard errors from the International Consortium for Blood Pressure GWAS (ICBP), which did not include the UK Biobank[4] (**Method S2**). The summary statistics for variants associated with type 2 diabetes were extracted from the GWAS, including 21,147 type 2 diabetes cases and 434,460 controls from the European subset of UK Biobank participants.[2] In this GWAS study, type 2 diabetes outcomes were defined using UK Biobank self-reports of the disease and ICD-10 diagnostic codes, and analysis was controlled for age and sex, population stratification, relatedness, and polygenic effect.[2] We used a two-sample Mendelian randomisation framework to estimate the effect using a random-effect inverse variance weighted method and applied several sensitivity and positive control analyses (**Method S3**).

Method S2. Details of the International Consortium for Blood Pressure genome-wide association study

To assess the causal association between blood pressure and risk of type 2 diabetes, we applied a two-sample Mendelian randomisation framework, in which the summary statistics from the International Consortium for Blood Pressure genome-wide association study (ICBP GWAS) were used for the analysis. ICBP is a genome-wide association meta-analysis, including about 200,000 participants from European countries, and its estimations were adjusted for sex, age, age-squared, body-mass index, within-cohort stratification, and also for blood pressure-lowering medication use.[4] The ICBP analyses were conducted using linear regression model and combined across studies using inverse-variance weighted meta-analysis.[4]

Method S3. Details of statistical methods and sensitivity analyses for Mendelian randomisation study

Two Sample Mendelian randomisation

The summary estimations of variants-exposure and variants-outcome were harmonised before conducting the statistical analysis.[3,5] The inverse-variance weighted method has been used as the main method and assumes that either all the instruments are valid or any horizontal pleiotropy is balanced.[6] We applied various Mendelian randomisation methods with different assumptions as sensitivity analyses to check the robustness and reliability of our findings:

We employed the weighted median method [7], which is consistent if at least 50% of the weight comes from valid instrumental variables.[8] The Mendelian Randomisation Pleiotropy RESidual Sum and Outlier (MR-PRESSO) method was used to test and, if needed, to correct for any possible horizontal pleiotropic outliers in the analysis.[9] The MR-Egger regression method was used to assess the presence of pleiotropy.[10] Although MR-Egger method is a worthwhile sensitivity analysis for detecting pleiotropy, it is susceptible to outlier genetic variants.[11] Therefore, we calculated Cook's distance measure [11,12] to detect the outlier variants, and then re-ran the MR-Egger analysis after removing the outlier variants. Robust Adjusted Profile Score (RAPS) estimator is robust to systematic and idiosyncratic pleiotropy and is recommended for complex traits and diseases.[13] MRMix method provides unbiased estimation in the presence of a large number of invalid genetic instruments. A methodological study suggested that MRMix produces more robust estimation compared to other conventional approaches.[14] Finally, we used Steiger filtering to remove genetic variants that are likely associated with diabetes through other causal pathways other than blood pressure.[15] We examined the heterogeneity of the estimates by using a scatter plot and applying Cochran's Q test.[16] We also assessed the probable directional pleiotropy using a funnel plot similar to that being used to assess for publication bias in meta-analysis.[16] A leave-one-out sensitivity analysis was conducted by removing a single variant from the analysis in turn. The fluctuation of the estimates in response to excluding each variant reflects the possibility of an outlier variant in the causal estimation. The 'MendelianRandomization' and 'TwoSampleMR' packages for R were used to implement the Mendelian randomisation analyses.[17,18]

The genome-wide association studies with blood pressure as phenotype routinely adjust for the effect of body mass index. [1,4] Using the estimates from body mass index-adjusted genome-wide association studies to conduct Mendelian randomisation could introduce collider bias. Therefore, we explored whether the identified causal association is driven by body mass index using unadjusted blood pressure estimations and by including body mass index as a phenotype in multivariable Mendelian randomisation. The UK Biobank dataset was used to derive the unadjusted estimates.[19] We used multivariable Mendelian randomisation

through the inverse-variance weighted method [20,21] to calculate adjusted versus unadjusted causal estimations.

Additionally, we tested the validity of the analysis by examining the effect between systolic blood pressure and coronary heart disease, myocardial infarction, and ischemic stroke as positive control outcomes. For this analysis, we utilised the same genetic variants for systolic blood pressure, but the variants-outcome association was extracted from independent genome-wide association studies.[22,23]

In a sensitivity analysis to further replicate the findings using different genome-wide association data, we extracted variants-outcome estimations from stage 1 of the DIAbetes Genetics Replication And Meta-analysis (DIAGRAM) Consortium.[24] The stage 1 of DIAGRAM consisted of 12,171 diabetes cases and 56,862 controls across 12 genome-wide association studies of individuals of European descent. In DIAGRAM, each genetic variant with a minor allele frequency of >1% passing quality control was tested for association with diabetes under an additive model.

One-sample Mendelian randomisation

To further replicate the result of two-sample Mendelian randomisation through a different framework, we followed a one-sample Mendelian randomisation approach using individual participant data from the UK Biobank. We used the UK Biobank data, which is a large prospective cohort study that included 502,602 participants aged 40 to 69 years, recruited between 2006 and 2010 from 22 assessment centres across the United Kingdom. Details of the UK Biobank design have been published elsewhere.[25,26] UK Biobank genotype data were imputed with IMPUTE4 using the Haplotype Reference Consortium and the UK10K + 1000 Genomes panel [27] to identify ~96 million variants for 487,381 participants. We excluded 55,208 individuals who were not white British, had a variant call rate <98% and were outliers based on heterozygosity. Finally, we included 432,173 participants in the one-sample Mendelian randomisation study. We built a weighted polygenic risk score as an instrumental variable for systolic blood pressure using independent genetic variants (linkage disequilibrium $r^2 < 0.05$) with minor allele frequency > 0.01 and $P < 5\times10^{-8}$ at a genome-wide level. Overall, 246 genetic variants were selected, all with imputation quality > 0.9 that have been shown to be associated with systolic blood pressure in a genome-wide association meta-analysis, including over one million participants of European ancestry.[1] To build a genetic risk score, first, each variant was recoded additively (0, 1, and 2) according to the number of alleles that decrease the log beta of systolic blood pressure. Then, each variant was weighed according to the regression coefficient obtained from the genome-wide association meta-analysis to give more weight to variants with stronger effects.[28] A weighted genetic risk score was constructed using the following formula: $(\beta_1\times\text{variant}_1)+(\beta_2\times\text{variant}_2)+\cdots(\beta_n\times\text{variant}_n)$, where βi was the regression coefficient associated with variant_i and obtained from the genome-wide association study. Additionally, we replicated the one-sample Mendelian randomisation in another sensitivity analysis. In this sensitivity analysis, to build a new genetic

risk score, we selected 370 genetic variants that have been reported to be associated with systolic blood pressure (linkage disequilibrium $r^2 < 0.05$, minor allele frequency > 0.01 and $P < 5\times10^{-8}$ at a genome-wide level which passed quality control) in the final ICBP genome-wide association dataset included 77 cohorts (n = 299,024, no overlap with UK Biobank). The instrumental variable analysis was performed using an adjusted, two-stage predictor substitution method that used the unweighted genetic risk score as an instrument variable.

Method S4. The genetic approaches used to replicate the effect of each antihypertensive drug class using genetic analysis.

Blood pressure-lowering drug class effects can be predicted through variants in genes that encode receptors related to their mechanism of action. By way of example, beta-blockers, as a sympatholytic class of drugs, work by inhibiting the activation of the beta-adrenergic receptors with adrenaline and noradrenaline, thereby, reducing heart rate, myocardial contractility, and cardiac output.[29] In the same way, ADRB1 is a gene that encodes the beta-1 adrenergic receptor present in cardiomyocytes and in the heart conduction system, which plays a role in heart rate and myocardial contractility. Therefore, genetic variants in the ADRB1 gene associated with systolic blood pressure can be used as a proxy for treatment effect for beta-blockers and thus help assess the effect of that drug class on the outcome of interest.[30] For this complementary analysis, the genetic variants suggested by Gill et al.,[31] and Walker et al.,[32] were considered to estimate the effect of the blood pressure-lowering drug classes. Further details on the selection of the candidate genetic variants for each class of drugs are described in the below section. Two-sample Mendelian randomisation, through the random-effect inverse-variance weighted approach, was used for statistical analysis. We used coronary heart disease as a positive control to compare the estimates with an outcome in which there is well-established evidence from RCTs, particularly for the effect of each class of antihypertensive.[33] The same GWAS studies described earlier were also utilized for this stage of the analysis.[2,4]

We were interested in selecting the approach that has a high statistical power and provides precise estimation for each drug class. The following steps were taken for selection:

a) For validation purposes and as a positive control outcome, we considered coronary heart disease (CHD) as the outcome of interest throughout this analysis because there is strong evidence for the protective effect of major classes of antihypertensives on the risk of CHD.[34–36]
b) The effect of each class of antihypertensive drug was assessed first using the placebo-controlled trials to provide trial-based estimation for the effect of each class versus placebo and then using Mendelian randomisation analysis through genetic variants reported by Gill et al.[37] and Walker et al.[38], separately.
c) For each class of drug, we selected the better performing approach (Gill or Walker method) if the estimated effect size met the two predefined criteria: 1) the effect size (point estimation) should be in the same direction as the estimation from placebo-controlled trials; 2) the estimates to have higher precision (narrower confidence intervals).
d) **Nested Figure i** shows the final selection. We selected the genetic variants for ACEIs/ARBs and thiazide diuretic from the Walker et al.[38] and beta-blockers and calcium channel blockers from the Gill et al.[37] The characteristics of genetic variants selected for each class of drugs are shown in the **Nested Table ii**.

Nested Figure i. Comparison of the effect of major antihypertensive drug classes on coronary heart disease as positive control outcome, using individual participant data meta-analysis of randomised placebo-controlled clinical trials and Mendelian randomisation

Blue squares represent the point estimation and size of squares is the same. The horizontal solid lines represent 95% confidence intervals. RR indicate hazard ratio in individual participant data meta-analysis and odds ratio in Mendelian randomisation; ACEIs/ARBs: angiotensin-converting enzyme inhibitors/angiotensin II receptor blockers

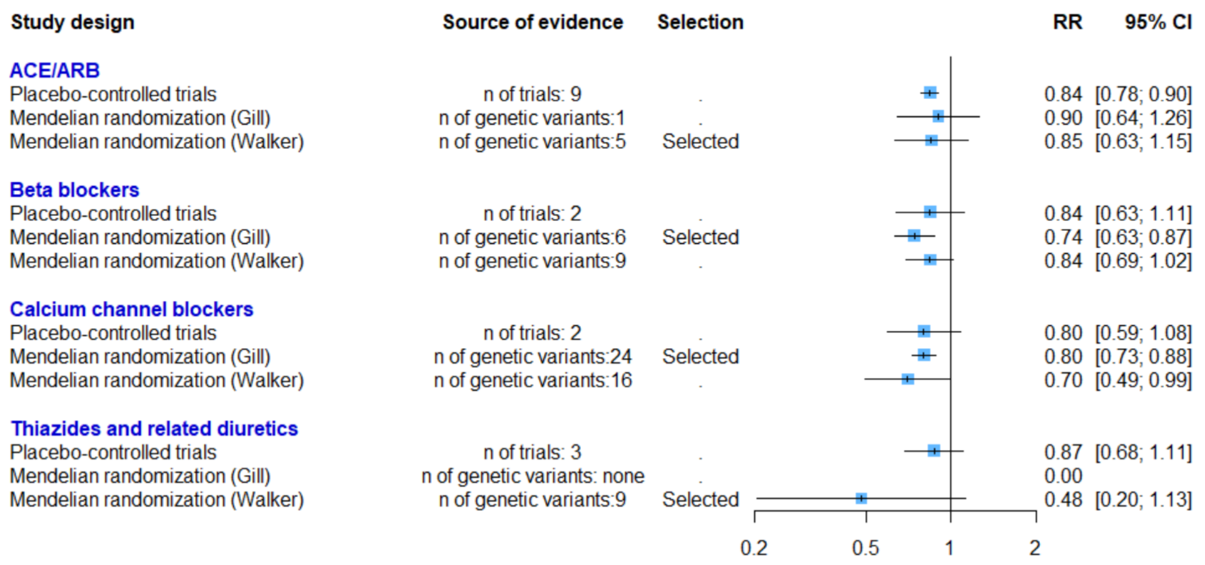

Nested Table ii. The characteristics of genetic variants selected to assess the effect of major antihypertensive drug classes on the risk of new-onset type 2 diabetes, using Mendelian randomisation approach.

Chromosome (GRCh37)	SNP	allele1	allele2	freq1	Effect	SE	p-value	Total sample size
**ACEIs/ARBs**								
3:148913426	rs118123032	t	c	0.0332	-0.153	0.1381	0.2677	273115
3:149370293	rs79387447	a	g	0.0145	0.0148	0.2263	0.9477	273295
3:148485148	rs80350379	t	c	0.0207	0.0982	0.1937	0.6121	272143
3:11652673	rs9829399	t	c	0.1172	0.1489	0.0716	0.03745	278480
17:61550729	rs4968783	a	c	0.6091	-0.2927	0.0472	5.54E-10	285133
**Beta-blockers**								
10:115707298	rs11196549	a	g	0.0421	0.7923	0.1255	2.75E-10	279594
10:115721364	rs460718	a	g	0.3222	-0.1832	0.0497	0.000226	279594
10:115788094	rs11196597	a	g	0.1379	0.2439	0.0716	0.000664	278589
10:115800294	rs17875473	t	c	0.0944	0.2345	0.0844	0.005472	279595
10:115805056	rs1801253	c	g	0.7305	0.4394	0.0524	5.08E-17	279594
10:115826508	rs4359161	a	g	0.1822	-0.2376	0.0592	6.09E-05	279593
**Calcium channel blockers**								
12:2434419	rs2239046	a	g	0.6788	0.2237	0.0483	3.61E-06	287243
12:2514270	rs714277	t	c	0.2837	0.2199	0.0503	1.24E-05	287245
10:18334521	rs2488136	a	g	0.2803	0.1453	0.0513	0.004658	279594
10:18440444	rs1888693	a	g	0.3472	0.3351	0.0482	3.57E-12	277475
10:18457722	rs16916914	t	c	0.9604	-0.4955	0.1192	3.22E-05	278849
10:18459450	rs7076319	a	g	0.7277	-0.2133	0.0516	3.56E-05	278479

10:18481737	rs61278674	a	g	0.911	-0.1719	0.0865	0.0469	278588
10:18514561	rs1779209	t	c	0.296	0.1356	0.0514	0.00836	270873
10:18553968	rs10828399	a	g	0.5323	-0.1507	0.0459	0.001027	279593
10:18592450	rs10828452	a	t	0.7949	0.2464	0.061	5.32E-05	278589
10:18627285	rs10828542	a	g	0.6157	0.143	0.0475	0.002622	279595
10:18678987	rs12780039	c	g	0.1201	0.1752	0.072	0.01497	279592
10:18695681	rs112133583	t	c	0.0266	-0.4123	0.1724	0.0168	278594
10:18710991	rs11014170	a	g	0.0234	-0.6098	0.1781	0.000618	273573
10:18727901	rs7923191	a	g	0.7884	-0.3403	0.0572	2.64E-09	278479
10:18727959	rs12258967	c	g	0.7104	0.5426	0.0529	1.06E-24	278590
10:18729855	rs72786098	a	g	0.0291	-0.3976	0.1472	0.006913	278480
10:18755664	rs1998822	a	g	0.7272	-0.1349	0.0529	0.01072	268756
10:18790727	rs4748474	a	g	0.5273	0.1149	0.0467	0.0138	271333
12:49209340	rs150857355	c	g	0.0213	1.0616	0.1906	2.56E-08	272725
3:53558012	rs3821843	a	g	0.6838	0.331	0.0524	2.61E-10	277474
3:53605712	rs114987861	a	g	0.0305	0.395	0.1472	0.007298	278479
3:53612327	rs113210396	t	g	0.0445	-0.3563	0.1293	0.005856	278589
3:53734443	rs7340705	t	c	0.6684	-0.1929	0.0485	7.08E-05	279594
**Thiazide diuretics**								
10:78695467	rs10762738	a	g	0.5005	0.0565	0.0469	0.2278	261609
15:26818362	rs8030011	a	g	0.1342	0.0187	0.0665	0.779	287242
15:27722954	rs140443467	a	g	0.9691	-0.0779	0.1645	0.6357	269648
15:47906718	rs12914000	t	c	0.8366	0.0882	0.0636	0.1658	286240
4:45844166	rs139787011	a	g	0.9834	0.1814	0.2274	0.425	257608
4:45956676	rs7699135	t	c	0.8659	-0.0591	0.0684	0.3876	279594
5:160335398	rs13188637	a	g	0.5052	-0.0181	0.0461	0.694	276577
5:161908897	rs10076365	a	g	0.8301	-0.0443	0.0616	0.4717	275462
8:87064009	rs62509890	a	g	0.8927	-0.134	0.0757	0.07688	279595

Supplementary Tables

Table S1. Selected published reports on the effect of blood pressure lowering per se and specific drug class effect on risk of new-onset type 2 diabetes.

Study	Publication date	Design	Study name	Size	Time of follow-up	Exposure in cohort studies or trial arms in RCTs	Finding	Conclusion	Comment
**Studies investigating the effect of blood pressure lowering per se on risk of type 2 diabetes**									
1) Meta-analysis of observational studies									
Emdin CA [39]	2015	-Observational cohort<br>- Meta-analysis of observational cohort studies	Clinical Practice Research Datalink (CPRD)	Observational cohort: 4.1 million<br>Meta-analysis: 30 studies with 285,664 participants and 17,388 incident diabetes events	A median follow-up of 6.8 years	Systolic BP per 20 mmHg increase	**Observational cohort**: 20 mmHg higher systolic BP was associated with a 58% higher risk of new-onset diabetes (hazard ratio 1.58; 95% CI 1.56 to 1.59)<br>**Meta-analysis**: The pooled relative risk of diabetes for a 20 mmHg higher usual systolic BP across studies was 1.77 (1.53 to 2.05).	Elevated BP was associated with an increased risk of new-onset diabetes.	- The largest ever observational analysis<br>- Observational study could not confirm the causal association
1) Individual randomised controlled trial									
Roumie CL [40]	2020	Randomised clinical trial	The Systolic Blood Pressure Intervention Trial (SPRINT)	8,380	3 years	More intense vs less intense treatment	Adjusted hazard ratio for incidence of diabetes was 1.19 (95% CI, 0.95–1.49)	No clear effect on risk of diabetes.	- Short length of follow up and not placebo-controlled<br>- Information about the combination of antihypertensive drugs used in each arm was limited, and hence the study was unable to separate the effects of BP reduction from varying drug classes off-target effect
2) Mendelian randomisation									
Sun D [41]	2019	Mendelian randomisation	UK Biobank	318,664	NA	Genetically determined hypertension	Genetically determined hypertension has no relationship with diabetes (odds ratio 0.96 [CI 95% 0.88 to 1.04])	No causal association	The lack of association in this study may be due to weak instrument bias because summary estimations for variants-exposure and variants-outcome were derived from the same population (i.e., UK biobank). Also, categorizing a continuous variable such as blood pressure to binary hypertension reduces the statistical power to detect any causal association
Aikens RC [42]	2017	Mendelian randomisation	NA	Summary statistics from GWAS meta-analysis	NA	Genetically determined higher systolic BP	2% increase in the risk of diabetes per 1 mmHg genetically determined higher systolic BP (odds ratio 1.02, 95% CI 1.01 to 1.03)	Elevated systolic BP was associated with an increased risk of diabetes	This study was based on only 28 genetic variants associated with systolic BP, however the findings are broadly consistent with our result
Zhu Z [43]	2018	Mendelian randomisation	NA	Summary statistics from GWAS meta-analysis	NA	Genetically determined higher systolic BP	- Analysis based on GWAS meta-analysis of two community-based studies (GERA and UKB) showed no association (odds ratio 1.07, 95% CI 0.89 to 1.29)<br>- Analysis based on published independent case-control studies revealed significant finding (odds ratio 1.46, 95% CI 0.13 to 1.89)	Inconsistent findings based on different dataset	Given the small number of instruments used, the analysis likely to be limited in statistical power
**Studies investigating the effect of antihypertensives on risk of type 2 diabetes**									
1) Observational studies									
Gress TW [44]	2000	Prospective cohort study	The Atherosclerosis Risk in Communities (ARIC)	12,550	6 years	Antihypertensive medications use in people without diabetes at baseline	- Thiazide diuretics were not significantly associated with greater risk of the subsequent development of diabetes (hazard ratio, 0.91; 95% CI, 0.73 to 1.13)<br>- ACEI were significantly not associated with greater risk of the subsequent development of diabetes (hazard ratio, 0.98; 95% CI, 0.72–1.34)<br>- Calcium channel blockers were not associated with a significantly greater risk of the subsequent development of diabetes hazard ratio 1.17, 95% CI 0.83 to 1.66)<br>- Beta-blocker increased the risk of new-onset diabetes (hazard ratio 1.28, 95% CI 1.04 to 1.57)	Beta-blockers were associated with an increased risk of diabetes, but for other classes no significant effect was found	Observational studies are prone to confounding and reverse causation and cannot confirm the effect of drug classes
2) Individual randomised controlled trial									
Fletcher AE [45]	1991	Randomised clinical trial	The European Working Party on High Blood Pressure	840	NA	Diuretics vs placebo	No significant effect on new-onset diabetes participant (risk ratio 1.47, 95% CI 0.84 to 1.57)	BP lowering treatment with diuretics had no significant effect on the incidence of	The sample size and number of events were comparatively small

			in the Elderly (EWPHE)					diabetes in elderly hypertensive patients	
Savage PJ [46]	1998	Multicenter, randomised, double-blind, placebo-controlled clinical trial	The Systolic Hypertension in the Elderly Program (SHEP)	4,736	3 years	Thiazide diuretic or beta blockers vs placebo	New cases of diabetes were reported by 8.6% of the participants in the active treatment group and 7.5% of the participants in the placebo group (risk ratio 1.14, 95% CI 0.90 to 1.45)	No significant increase in risk of diabetes	The sample size and number of events were comparatively small
Cooper-DeHoff R [47]	2006	Randomised clinical trial	International Verapamil SR-Trandolapril Study (INVEST)	16,176	2.8 years	Calcium channel blockers versus beta-blockers	Risk of new-onset diabetes was lower in patients who took calcium channel blockers than beta-blocker (hazard ratio 0.85, 95% CI 0.76 to 0.95).	Beta-blockers compared with calcium channel blockers increased the risk of diabetes	Given the number of diabetes events (25 cases in calcium channel blockers and 30 cases in beta-blockers groups), the findings were not robust. No insights on drug vs placebo effects.
3) Meta-analyses of trials									
Elliott WJ [48]	2007	Network meta-analysis of clinical trials	NA	22 trials with 143,153 participants	NA	Antihypertensive agents	Considering the diuretics as the comparator group, the odds ratios were: 0.57 (95% CI 0.46 to 0.72) for ARB, 0.67 (0.56 to 0.80) for ACEI, 0.75 (0.62 to 0.90) for Calcium channel blockers, 0.77 (0.63 to 0.94) for placebo and 0.90 (0.75 to 1.09) for beta-blockers.	ARBs and ACEIs are least associated with the risk of new-onset diabetes, followed by Calcium channel blockers, placebo, beta-blockers, and thiazide diuretic. All drug classes appeared to have a more favourable effect than diuretics	Similar to our network meta-analysis, a high proportion of evidence in this study was estimated through indirect comparisons. With the main comparisons being against diuretics (which are unlikely to have a neutral effect on diabetes), the interpretation and clinical implications have remained elusive. A sensitivity analysis reported effects against placebo but the confidence intervals for some comparisons were wide, leading to inconclusive estimates for ACEIs and beta-blockers.
4) Mendelian randomisation									
No study									

BP: blood pressure, RCT: randomised controlled trial, CI: confidence intervals, GWAS: genome wide association study, ACEI: Angiotensin-converting-enzyme inhibitors, ARB: angiotensin II receptor blocker, NA: not applicable

Table S2. Diagnostic criteria for the definition of type 2 diabetes in each trial.

		Treatment		Comparator	
**Trial name**	**New-onset type 2 diabetes definition**	**Events**	**Total**	**Events**	**Total**
ACTIVE I [49]	Adverse event report	196	3614	213	3617
ALLHAT [50]	Fasting blood glucose of ≥ 7.0 mmol/L (126 mg/dL)	1374	9719	1810	17393
ANBP [51]	Adverse event report	14	1717	13	1704
ANBP2 [52]	Adverse event report	184	2817	127	2795
ASCOT-BPLA [53]	Fasting blood glucose of ≥ 7.0 mmol/L (126 mg/dL)	565	7032	792	6982
CAPPP [54]	Two determinations of fasting blood glucose of ≥ 6.7 mmol/L (120.6 mg/dL) according to the 1985 World Health Organization criteria.	380	5205	337	5154
CASEJ [55]	Self-reported diabetes or anti-diabetic agents	59	1293	38	1302
COLM [56]	Initial diagnosis by participating physicians and final ascertainment by the endpoint committee	15	1840	11	1844
COPE [57]	Adverse event report	20	956	69	1871
HIJ-CREATE [58]	Fasting blood glucose ≥ 7.0 mmol/L (126 mg/dL) or commencement of anti-diabetic agents and/or glycohemoglobin A1c ≥ 6.5%	7	645	18	624
HOPE [59]	Adverse event report	102	4645	155	4652
INSIGHT [60]	World Health Organization criteria	136	3154	176	3163
MOSES [61]	Adverse event report	11	416	19	433
NORDIL [62]	Adverse event report	249	5026	216	4980
ONTARGET [63]	Fasting blood glucose of ≥ 7.0 mmol/L (126 mg/dL)	323	5280	761	10717
PEACE [64]	Adverse event report	334	3417	399	3457
PROGRESS [65]	Adverse event report	80	2657	86	2685
STOP2 [66]	Two determinations of fasting blood glucose of ≥ 6.7 mmol/L (120.6 mg/dL)	97	1954	190	3923
SYSTEUR [67]	International Classification of Diseases, ninth revision	107	2165	78	2069
TRANSCEND [68]	Fasting blood glucose of ≥ 7.0 mmol/L (126 mg/dL)	205	1889	238	1905
VALUE [69]	Fasting blood glucose of > 7.8 mmol/L (140.4 mg/dL)	690	7649	845	7596
PROFESS [70]	Adverse event report	112	7108	136	7103

ACTIVE I: Atrial Fibrillation Clopidogrel Trial with Irbesartan for Prevention of Vascular Events; ALLHAT: Antihypertensive and Lipid Lowering Treatment to Prevent Heart Attack Trial; ANBP: Australian National Blood Pressure Study; ANBP2: Second Australian National Blood Pressure Study; ASCOT-BPLA: Anglo-Scandinavian Cardiac Outcomes Trial-Blood Pressure Lowering Arm; CAPPP: Captopril Prevention Project; CASE-J: Candesartan Antihypertensive Survival Evaluation in Japan Trial; COLM: Combination of OLMesartan study; COPE: Combination Therapy of Hypertension to Prevent Cardiovascular Events; HIJ-CREATE: Heart Institute of Japan Candesartan Randomized Trial for Evaluation in Coronary Artery Disease; HOPE: Heart Outcomes Prevention Evaluation; INSIGHT: International Nifedipine GITS study: Intervention as a Goal in Hypertension Treatment; MOSES: Morbidity and Mortality After Stroke, Eprosartan Compared With Nitrendipine for Secondary Prevention; NORDIL: Nordic Diltiazem Study; ONTARGET: Ongoing Telmisartan Alone and in Combination with Ramipril Global Endpoint Trial; PEACE: Prevention of Events with Angiotensin-Converting Enzyme Inhibition; PRoFESS: Prevention Regimen for Effectively Avoiding Second Strokes; PROGRESS: Perindopril Protection Against Recurrent Stroke Study; STOP Hypertension-2: Swedish Trial in Old Patients with Hypertension-2; Syst-Eur: Systolic Hypertension in Europe; TRANSCEND: Telmisartan Randomized Assessment Study in ACE Intolerant Subjects with Cardiovascular Disease; VALUE:Valsartan Antihypertensive Long-term Use Evaluation

Table S3. General characteristics of trials included in the individual patient data meta-analysis as well as Bayesian network meta-analysis.

Trial name	Design	Inclusion criteria	Exclusion criteria	Follow-up duration (years)	Trial arms		Incident diabetes cases†	Total participants	Diabetes event date¶
					Intervention*	Comparator			
ACTIVE I [49]	Placebo-controlled	Atrial fibrillation, ≥1 risk factor (age ≥75 years, on antihypertensive treatment, history of stroke, TIA or non-CNS embolism, LVEF <45%, PVD, or age 55-74 years with either CAD or diabetes)	Use of anticoagulant, peptic ulcer disease in past 6 months, history of intracerebral haemorrhage, thrombocytopenia, or mitral stenosis	4.1	ARB	Placebo	409	7,231	Available
ALLHAT [35]	Head-to-head	Age ≥55 years stage 1 or 2 hypertension plus ≥1 risk factor (MI or stroke >6 months previously, left ventricular hypertrophy, T2D, smoking, HDL <0.91 mmol/l), other atherosclerotic CVD	Symptomatic or hospitalisation for heart failure, LVEF <35%	4.8	Diuretic	ACEI, CCB or alpha-blocker	3,184	27,135	Available
ANBP [51]	Placebo-controlled	Age 30-69 years with mild hypertension (DBP 95-110 mmHg and SBP <200 mmHg)	Antihypertensive treatment in the past 3 months, recent angina or MI, stroke, hormone therapy, asthma, diabetes, gout, severe disease, tricyclic antidepressant use	3.6	Diuretic	Placebo	27	3,427	Available
ANBP2 [52]	Head-to-head	Age 65-84 years, SBP ≥160 mmHg or DBP ≥90 mmHg (if SBP≥140 mmHg), no recent CVD	Serious illness, plasma creatinine >221 μmol/l, malignant hypertension, dementia	4.1	Diuretic	ACEI	341	5,642	Available
ASCOT-BPLA [71]	Head-to-head	Age 40-79 years, untreated (SBP ≥160 or DBP ≥100 mmHg) or treated hypertension (SBP ≥140 or DBP ≥90 mmHg), ≥3 CVD risk factors (documented LVH, abnormal ECG, T2D, PAD, previous stroke or TIA, male sex, age ≥55 years, microalbuminuria or proteinuria, smoking, TC: HDL ≥6, family history of premature coronary heart disease	Previous MI, current treatment for angina, recent CeVD, fasting triglycerides >4.5 mmol/l, heart failure, arrhythmia, haematological or biochemical abnormality at screening	5.3	CCB-based (+ACEI)	BB-based (+ diuretic)	1,358	14,112	Available
CAPPP [54]	Head-to-head	Age 25-66 years, DBP ≥100 mmHg on two occasions	Secondary hypertension, serum creatinine >150 μmol/, a condition requiring BB treatment	5.8	BB and diuretic	ACEI	717	10,413	Available
CASEJ [55]	Head-to-head	Age 20-85 years, ≥1 high-risk factor: SBP ≥180 or DBP ≥110 mmHg, T2D, history of angina pectoris, MI, stroke, TIA >6 months before screening, LVH, proteinuria or serum creatinine ≥1.3 mg/100 ml, peripheral artery obstruction	BP ≥200/120 mmHg, T1D, heart failure, LVEF <40%, atrial fibrillation, cancer	3.1	CCB	ARB	97	2,685	Available
COLM [72]	Head-to-head	Age 65-84 years, hypertension (treated: BP ≥140/90 mmHg; untreated: BP ≥160/100 mmHg), CVD history or CVD risk factors (diabetes, dyslipidemia)	Secondary/malignant hypertension, recent major CVD, revascularisation, angina pectoris hospitalisation or severe heart failure, atrial fibrillation, hepatic or renal dysfunction	3.0	ARB and diuretic	ARB and CCB	26	3,779	Available
COPE [57]	Head-to-head	Age 40-85 years, BP ≥140/90 mmHg	SBP ≥200 or DBP ≥120 mmHg, secondary hypertension, diabetes, recent CVD or revascularisation, heart failure, atrial fibrillation/flutter, hepatic or renal dysfunction, congenital or rheumatic heart disease, cancer	3.6	CCB and ARB	CCB and diuretic or CCB and BB	89	2,827	Available
HIJ-CREATE [58]	Head-to-head	Age 20-80 years, CAD hospitalisation and hypertension (BP ≥140/90 mmHg or antihypertensive treatment use)	Secondary hypertension, recent AMI or CeVD, severe aortic valve stenosis, cardiomyopathy, serum creatinine >2 mg/dl, serum potassium >5 mmol/l, hepatic dysfunction, malignancy	4.0	ARB	non-ARB (including ACEI)	25	1269	Available
HOPE [59]	Placebo-controlled	Age ≥55 years, CAD, stroke, PVD or diabetes, plus ≥1 risk factor (hypertension, dyslipidemia, smoking, or documented microalbuminuria)	Heart failure, LVEF <40%, using ACEI or Vitamin E, uncontrolled hypertension, nephropathy, or recent MI or stroke	4.5	ACEI	Placebo	257	5,720	Unavailable
INSIGHT [60]	Head-to-head	Age 55-80 years, hypertensive (SBP ≥150 or DBP ≥95 mmHg, or SBP ≥160 mmHg), ≥1 other risk factor (TC ≥6.43 mmol/l, smoking, family history of premature MI, CAD, other CVD	None specified	2.8	Diuretic	CCB	312	5,015	Unavailable
MOSES [61]	Head-to-head	Hypertension requiring treatment, documented TIA, ischemic stroke or cerebral haemorrhage	Internal carotid artery occlusion or stenosis >70%, heart failure, age >85 years, on anticoagulant for cardiac arrhythmia, high-grade aortic or mitral valve stenosis, unstable angina	3.3	CCB	ARB	34	854	Available
NORDIL [62]	Head-to-head	Age 50-74 years, untreated hypertension (DBP ≥100 mmHg on two occasions); if previously treated, DBP ≥100 mmHg on two consecutive visits at one week apart during run-in period and no treatment was given	Age <50 or ≥70 years, bradycardia, secondary hypertension, atrial fibrillation, recent CeVD or MI, heart failure	4.2	BB and diuretic	CCB	465	10,154	Available
ONTARGET [73]	Head-to-head	CAD, PAD, CeVD or diabetes with end-organ damage	Heart failure, pericarditis, congenital heart disease, unexplained syncope, planned revascularisation <3 months of consent, uncontrolled hypertension, heart transplant, subarachnoid haemorrhage, renal artery disease, proteinuria, hepatic dysfunction,	4.8	ARB or ACEI	ACEI, ARB	1,088	16,008	Available

			volume, or sodium depletion, primary hyper-aldosteronism, hereditary fructose intolerance, other serious conditions						
PEACE [64]	Placebo-controlled	Age ≥50 years, documented CAD	Unstable angina, severe valvular heart disease, recent revascularisation, planned elective revascularisation, limited 5-year survival, serum creatinine >177 μmol/l, serum potassium >5.5 mmol/l	4.7	ACEI	Placebo	734	6,910	Available
PROGRESS [65]	Placebo-controlled	Stroke or TIA in the past 5 years	Indication or contraindication for ACEI	3.9	ACEI and/or diuretic	Placebo	168	5,344	Available
STOP2 [74]	Head-to-head	Aged 70-84 years, SBP ≥180 mmHg and/or DBP ≥105 mmHg	Not specified	4.5	BB and/or diuretic	ACEI and CCB	288	5,895	Available
SYSTEUR [75]	Placebo-controlled	Age ≥60 years, sitting SBP 160-219 mmHg, sitting DBP <95 mmHg, and standing SBP ≥140 mmHg	Secondary hypertension, retinal haemorrhage/papilledema, heart failure, dissecting aortic aneurysm, serum creatinine ≥180 μmol/l, recent severe nosebleeds, stroke or MI, dementia, disorders prohibiting standing position, severe CVD/non-CVD	2.6	CCB	Placebo	185	4,246	Available
TRANSCEND [68]	Placebo-controlled	Intolerant to ACEI and with established CAD, PVD, CeVD or diabetes with end-organ damage	Heart failure, valvular/cardiac outflow tract obstruction, pericarditis, congenital heart disease, unexplained syncope, recent revascularisation, SBP >160 mmHg, heart transplantation, subarachnoid haemorrhage, significant renal stenosis, renal or hepatic dysfunction	4.9	ARB	Placebo	454	3,808	Available
VALUE [69]	Head-to-head	Age ≥50 years, hypertension, CVD, CVD risk factors (male sex, age >50 years, diabetes, current smoking, high cholesterol, LVH, proteinuria, serum creatinine 150 to 265 μmol/l)	Renal artery stenosis, recent CAD or CeVD, severe hepatic disease or chronic renal failure, heart failure, on monotherapy with BB for CAD and hypertension	4.2	CCB-based	ARB-based	1,535	10,422	Unavailable
PROFESS [70]	Placebo-controlled	Age ≥55 years with ischemic stroke <90 days before randomisation (later modified to include age 50 to 54 years or had stroke 90 to 120 days before randomisation if with ≥2 additional risk factors: diabetes, hypertension, smoker, obesity previous CVD, end-organ damage or hyperlipidemia) and remained stable	Haemorrhagic stroke, severe disability after the qualifying stroke, contraindication to treatments	2.5	ARB	Placebo	248	14211	Available

* Treatment arm in head-to-head trials compared two or more drug classes defined based on the following predefined structure: the arm with the greater systolic blood pressure reduction was considered the intervention and the other(s) as the comparator.
† All patients with known diabetes diagnosis at baseline have been excluded.
¶ All trials without information for the time of diabetes occurrence were excluded from the individual patient data meta-analysis to assess the effect of blood pressure reduction and risk of diabetes.

BP: blood pressure; GFR: Glomerular filtration rate; DBP: diastolic blood pressure; CKD: chronic kidney disease; T2D: type 2 diabetes; CAD: coronary artery disease; CeVD: cerebrovascular disease; CVD: cardiovascular disease; HbA1c: glycated haemoglobin; SBP: systolic blood pressure; TIA: transient ischemic attack; PAD: peripheral artery disease; MI: myocardial infarction; CNS: the central nervous system; LVEF: left ventricular ejection fraction; HDL: high-density lipoprotein; ECG: electrocardiogram; TC: total cholesterol; LVH: left ventricular hypertrophy; T1D: type 1 diabetes; CCB: calcium channel-blocker; ACEI: angiotensin-converting enzyme inhibitor; ARB: angiotensin II receptor blocker; BB: beta blocker

ACTIVE I: Atrial Fibrillation Clopidogrel Trial with Irbesartan for Prevention of Vascular Events; ALLHAT: Antihypertensive and Lipid Lowering Treatment to Prevent Heart Attack Trial; ANBP: Australian National Blood Pressure Study; ANBP2: Second Australian National Blood Pressure Study; ASCOT-BPLA: Anglo-Scandinavian Cardiac Outcomes Trial-Blood Pressure Lowering Arm; CAPPP: Captopril Prevention Project; CASE-J: Candesartan Antihypertensive Survival Evaluation in Japan Trial; COLM: Combination of OLMesartan study; COPE: Combination Therapy of Hypertension to Prevent Cardiovascular Events; HIJ-CREATE: Heart Institute of Japan Candesartan Randomized Trial for Evaluation in Coronary Artery Disease; HOPE: Heart Outcomes Prevention Evaluation; INSIGHT: International Nifedipine GITS study: Intervention as a Goal in Hypertension Treatment; MOSES: Morbidity and Mortality After Stroke, Eprosartan Compared With Nitrendipine for Secondary Prevention; NORDIL: Nordic Diltiazem Study; ONTARGET: Ongoing Telmisartan Alone and in Combination with Ramipril Global Endpoint Trial; PEACE: Prevention of Events with Angiotensin-Converting Enzyme Inhibition; PRoFESS: Prevention Regimen for Effectively Avoiding Second Strokes; PROGRESS: Perindopril Protection Against Recurrent Stroke Study; STOP Hypertension-2: Swedish Trial in Old Patients with Hypertension-2; Syst-Eur: Systolic Hypertension in Europe; TRANSCEND: Telmisartan Randomized Assessment Study in ACE Intolerant Subjects with Cardiovascular Disease; VALUE: Valsartan Antihypertensive Long-term Use Evaluation

Table S4. Genetic variants selected as an instrumental variable for systolic blood pressure

(the information in this Table is extracted and modified from the main genome-wide association study by Evangelou et al.,) [1]

SNP *	Chromosome	Position (GRCh37)	Allele1	Allele2	Freq1†	Effect †	Standard error †	P-value†	Source *
rs3737801	1	27960832	c	g	0.9142	0.4246	0.0954	8.67E-06	Novel:one-stage design
rs11210029	1	41865293	a	g	0.625	-0.1608	0.0476	0.000728	Novel:one-stage design
rs11579440	1	49052423	t	c	0.8468	0.2794	0.0653	1.86E-05	Novel:one-stage design
rs10923038	1	88651771	a	c	0.6166	0.1279	0.0481	0.00781	Novel:one-stage design
rs76719272	1	156129796	t	c	0.1309	-0.2747	0.0727	0.00016	Novel:two-stage design
rs1043069	1	180859368	t	g	0.6225	0.2696	0.0478	1.72E-08	Novel:two-stage design
rs4651224	1	184585182	t	c	0.4518	0.144	0.047	0.002185	Novel:two-stage design
rs12042924	1	197297417	t	c	0.5202	-0.1372	0.0465	0.003202	Novel:two-stage design
rs33996239	1	203109801	t	c	0.0577	-0.427	0.1058	5.43E-05	Novel:two-stage design
rs7555285	1	209970355	c	g	0.7951	0.173	0.0565	0.002212	Novel:two-stage design
rs260508	1	2187085	t	g	0.6167	0.1696	0.0477	0.000377	Novel:two-stage design
rs2807337	1	22577371	t	c	0.3721	0.1938	0.0478	5.02E-05	Novel:two-stage design
rs4926499	1	249155909	c	g	0.8262	0.2922	0.0752	0.000102	Novel:two-stage design
rs79598313	1	27284913	t	c	0.0275	0.5126	0.1518	0.00073	Novel:two-stage design
rs839755	1	43856410	a	c	0.6224	-0.1877	0.047	6.55E-05	Novel:two-stage design
rs7514579	1	94051350	a	c	0.7765	0.3027	0.0559	6.16E-08	Novel:two-stage design
rs17396055	1	94730954	a	g	0.3317	-0.1525	0.049	0.001874	Novel:two-stage design
rs880315	1	10796866	t	c	0.652	-0.5218	0.0499	1.33E-25	Previously reported
rs4846049	1	11850365	t	g	0.3264	-0.4146	0.0489	2.44E-17	Previously reported
rs17367504	1	11862778	a	g	0.8444	0.7774	0.0639	4.81E-34	Previously reported
rs5068	1	11905974	a	g	0.9387	1.0914	0.0989	2.53E-28	Previously reported
rs3820068	1	15798197	a	g	0.7977	0.3361	0.0596	1.69E-08	Previously reported
rs7515635	1	42408070	t	c	0.4684	0.2382	0.0463	2.70E-07	Previously reported
rs10922502	1	89360158	a	g	0.6407	-0.2283	0.0483	2.32E-06	Previously reported
rs55732192	2	162278233	t	g	0.0962	-0.2807	0.0798	0.000433	Novel:one-stage design
rs6712203	2	165557318	t	c	0.3779	-0.1943	0.0477	4.57E-05	Novel:one-stage design
rs11694601	2	174949358	a	g	0.5927	-0.1422	0.047	0.00249	Novel:one-stage design

rs1837164	2	178716601	a	t	0.3753	0.186	0.0472	8.27E-05	Novel:one-stage design
rs296797	2	201102905	t	c	0.4142	0.2067	0.0467	9.68E-06	Novel:one-stage design
rs1047891	2	211540507	a	c	0.3243	-0.1647	0.0511	0.00127	Novel:one-stage design
rs10189186	2	53025757	a	g	0.5357	0.1752	0.0459	0.000135	Novel:one-stage design
rs28377357	2	112769721	a	g	0.3	-0.1588	0.0502	0.001572	Novel:two-stage design
rs6723509	2	122000745	t	c	0.8617	0.2553	0.0677	0.000162	Novel:two-stage design
rs72844590	2	138421227	t	g	0.1441	0.0856	0.0692	0.2158	Novel:two-stage design
rs79523138	2	161368213	a	g	0.8849	-0.3083	0.0749	3.83E-05	Novel:two-stage design
rs6739913	2	185033065	a	g	0.7095	-0.1523	0.0506	0.002638	Novel:two-stage design
rs28558491	2	187816321	t	c	0.7362	-0.1935	0.0531	0.000265	Novel:two-stage design
rs67720684	2	18975439	a	c	0.2295	0.0834	0.0546	0.1269	Novel:two-stage design
rs12694277	2	213188795	t	c	0.2914	-0.219	0.051	1.77E-05	Novel:two-stage design
rs1044822	2	230629138	t	c	0.142	-0.2655	0.0657	5.38E-05	Novel:two-stage design
rs139354822	2	242344695	t	c	0.9675	0.4794	0.1554	0.002042	Novel:two-stage design
rs35590893	2	43716933	a	g	0.2716	-0.1215	0.0515	0.01822	Novel:two-stage design
rs6545155	2	50429861	t	c	0.7852	0.2182	0.0559	9.56E-05	Novel:two-stage design
rs2920899	2	55279681	t	g	0.7851	0.1653	0.0573	0.003886	Novel:two-stage design
rs72816333	2	60096560	a	t	0.8277	0.254	0.0606	2.79E-05	Novel:two-stage design
rs2300481	2	66782467	t	c	0.3886	0.2043	0.0472	1.50E-05	Novel:two-stage design
rs1446468	2	164963486	t	c	0.4512	-0.487	0.0468	2.26E-25	Previously reported
rs6712094	2	165043460	a	g	0.7296	0.42	0.0525	1.17E-15	Previously reported
rs6749447	2	169041386	t	g	0.7323	-0.067	0.0523	0.2008	Previously reported
rs6434404	2	191494411	a	g	0.3247	0.2228	0.0498	7.53E-06	Previously reported
rs1344653	2	19730845	a	g	0.4961	-0.1568	0.0456	0.00058	Previously reported
rs55780018	2	208526140	t	c	0.548	-0.3278	0.0488	1.85E-11	Previously reported
rs2972146	2	227100698	t	g	0.6362	0.2486	0.0476	1.76E-07	Previously reported
rs55701159	2	25139596	t	g	0.887	0.2999	0.0742	5.27E-05	Previously reported
rs1275988	2	26914364	t	c	0.6055	-0.5157	0.0466	1.83E-28	Previously reported
rs9678851	2	27887034	a	c	0.559	-0.1135	0.0474	0.01662	Previously reported
rs7562	2	28635740	t	c	0.5297	0.1555	0.047	0.00093	Previously reported

rs13420463	2	37517566	a	g	0.7775	0.2751	0.0555	7.19E-07	Previously reported
rs262986	3	183435713	a	g	0.4712	-0.2288	0.0468	1.01E-06	Novel:one-stage design
rs1882289	3	114461208	a	g	0.8814	-0.2919	0.0708	3.78E-05	Novel:two-stage design
rs9875380	3	132780356	t	c	0.4619	-0.2472	0.0457	6.21E-08	Novel:two-stage design
rs863930	3	135949737	t	g	0.4671	-0.191	0.046	3.28E-05	Novel:two-stage design
rs78151625	3	158316726	t	c	0.831	-0.222	0.0618	0.000329	Novel:two-stage design
rs189267552	3	20073193	a	t	0.0141	-0.7415	0.2166	0.000618	Novel:two-stage design
rs12638085	3	30405936	a	t	0.3525	0.2514	0.0486	2.30E-07	Novel:two-stage design
rs6788984	3	41107173	a	g	0.858	0.3015	0.066	4.92E-06	Novel:two-stage design
rs6774721	3	49381898	a	g	0.1465	-0.2171	0.0689	0.001627	Novel:two-stage design
rs9857362	3	74710462	a	c	0.5249	0.1736	0.0473	0.000241	Novel:two-stage design
rs347591	3	11290122	t	g	0.6625	0.2842	0.0489	6.31E-09	Previously reported
rs11128722	3	14958126	a	g	0.5628	-0.2518	0.047	8.53E-08	Previously reported
rs143112823	3	154707967	a	g	0.076	-0.4019	0.0949	2.29E-05	Previously reported
rs3097937	4	124794644	a	t	0.8075	0.2388	0.0587	4.77E-05	Novel:one-stage design
rs6823767	4	151295085	t	c	0.7227	-0.1566	0.0528	0.003027	Novel:one-stage design
rs7439567	4	138464842	t	c	0.4157	0.245	0.0474	2.39E-07	Novel:two-stage design
rs17035181	4	157678511	t	g	0.8549	0.2169	0.0653	0.000898	Novel:two-stage design
rs2610990	4	18008232	a	g	0.2693	-0.2325	0.0523	8.86E-06	Novel:two-stage design
rs231708	4	2694773	c	g	0.6983	-0.2643	0.0499	1.19E-07	Novel:two-stage design
rs12511987	4	46595623	t	g	0.8224	-0.2456	0.0614	6.26E-05	Novel:two-stage design
rs1347345	4	95938386	a	g	0.6206	-0.1645	0.0478	0.000583	Novel:two-stage design
rs13112725	4	106911742	c	g	0.7682	0.397	0.0557	1.01E-12	Previously reported
rs2291435	4	38387395	t	c	0.5248	-0.2419	0.0463	1.74E-07	Previously reported
rs2014912	4	86715670	t	c	0.1515	0.5122	0.0644	1.80E-15	Previously reported
rs1650911	5	141740620	c	g	0.7619	0.2465	0.0584	2.42E-05	Novel:one-stage design
rs12153395	5	179411477	a	g	0.1133	-0.2602	0.0764	0.000661	Novel:one-stage design
rs4957026	5	361148	a	g	0.3503	0.2214	0.0497	8.29E-06	Novel:one-stage design
rs6875372	5	64079015	a	t	0.5154	0.2228	0.0459	1.18E-06	Novel:one-stage design
rs1871190	5	97953719	t	g	0.3472	0.1658	0.0495	0.000805	Novel:one-stage design

rs62373688	5	127352807	a	t	0.1259	0.3593	0.0714	4.76E-07	Novel:two-stage design
rs10069690	5	1279790	t	c	0.2583	0.3827	0.0627	1.03E-09	Novel:two-stage design
rs702395	5	140086677	t	c	0.4369	0.2367	0.0468	4.31E-07	Novel:two-stage design
rs74774746	5	33411769	c	g	0.2639	-0.1177	0.0541	0.02953	Novel:two-stage design
rs13179413	5	55868097	t	c	0.2775	0.1383	0.0544	0.01098	Novel:two-stage design
rs3121685	5	65662133	t	c	0.4815	-0.2015	0.046	1.17E-05	Novel:two-stage design
rs246973	5	68007803	t	c	0.2833	0.1984	0.0509	9.60E-05	Novel:two-stage design
rs709668	5	96174186	a	g	0.1965	-0.2755	0.0576	1.72E-06	Novel:two-stage design
rs10077885	5	114390121	a	c	0.498	-0.2465	0.0484	3.54E-07	Previously reported
rs1008058	5	122435627	a	g	0.1183	0.3142	0.0766	4.12E-05	Previously reported
rs13359291	5	122476457	a	g	0.1654	0.4005	0.062	1.06E-10	Previously reported
rs6595838	5	127868199	a	g	0.2891	0.2361	0.0507	3.14E-06	Previously reported
rs11953630	5	157845402	t	c	0.3694	-0.4463	0.0501	5.15E-19	Previously reported
rs1421811	5	32714270	c	g	0.6116	0.4743	0.0477	2.46E-23	Previously reported
rs1173771	5	32815028	a	g	0.3976	-0.5227	0.0468	6.04E-29	Previously reported
rs10059921	5	87514515	t	g	0.0846	-0.3732	0.0919	4.89E-05	Previously reported
rs7765526	6	147713764	a	g	0.4682	0.2317	0.047	8.11E-07	Novel:one-stage design
rs9449350	6	82281417	t	c	0.673	-0.2333	0.0488	1.72E-06	Novel:one-stage design
rs9401090	6	119113317	t	c	0.7538	0.2512	0.054	3.32E-06	Novel:two-stage design
rs10782230	6	126228512	a	g	0.4907	0.2787	0.0459	1.27E-09	Novel:two-stage design
rs9885632	6	131311909	t	c	0.7338	0.245	0.052	2.42E-06	Novel:two-stage design
rs7763294	6	140383733	t	g	0.3169	-0.2059	0.0493	2.95E-05	Novel:two-stage design
rs2745599	6	1613686	a	g	0.5476	0.2128	0.0513	3.30E-05	Novel:two-stage design
rs9368222	6	20686996	a	c	0.2767	0.1639	0.0511	0.001338	Novel:two-stage design
rs6911827	6	22130601	t	c	0.4623	0.152	0.0473	0.001295	Previously reported
rs2270860	6	43270151	t	c	0.3092	0.2966	0.05	3.09E-09	Previously reported
rs10948071	6	43280713	t	c	0.5993	-0.2074	0.0465	8.13E-06	Previously reported
rs1563788	6	43308363	t	c	0.2937	0.3062	0.0501	9.79E-10	Previously reported
rs78648104	6	50683009	t	c	0.8985	-0.3571	0.083	1.69E-05	Previously reported
rs35410524	6	96885405	t	c	0.1917	0.2999	0.0588	3.38E-07	Previously reported

rs1870735	7	155744303	c	g	0.4548	0.2137	0.0486	1.08E-05	Novel:one-stage design
rs12979	7	24738164	c	g	0.8745	0.2241	0.0693	0.001227	Novel:one-stage design
rs34072724	7	130432469	a	g	0.4828	-0.1967	0.0465	2.37E-05	Novel:two-stage design
rs12703989	7	140238048	a	g	0.494	0.1026	0.0474	0.03035	Novel:two-stage design
rs11771693	7	150050111	a	g	0.6743	0.169	0.0502	0.000757	Novel:two-stage design
rs10274928	7	28142088	a	g	0.4932	0.1644	0.0475	0.000538	Novel:two-stage design
rs10233127	7	30933453	a	t	0.1087	0.2638	0.0805	0.001051	Novel:two-stage design
rs6593297	7	56122058	a	t	0.3178	0.0982	0.0523	0.06052	Novel:two-stage design
rs6963105	7	75097488	a	g	0.4432	-0.2035	0.0531	0.000127	Novel:two-stage design
rs848445	7	77572461	t	c	0.2821	-0.2067	0.0528	9.04E-05	Novel:two-stage design
rs17477177	7	106411858	t	c	0.7906	-0.5642	0.0564	1.60E-23	Previously reported
rs4728142	7	128573967	a	g	0.4383	-0.2155	0.0467	3.91E-06	Previously reported
rs13238550	7	131059056	a	g	0.3909	0.1695	0.0472	0.000329	Previously reported
rs10224002	7	151415041	a	g	0.7186	-0.2375	0.0525	5.99E-06	Previously reported
rs6969780	7	27159136	c	g	0.0961	0.3697	0.0793	3.12E-06	Previously reported
rs142449193	8	102750597	t	c	0.0491	-0.4354	0.112	0.000102	Novel:one-stage design
rs4875958	8	1721090	a	g	0.7099	0.2209	0.0515	1.83E-05	Novel:one-stage design
rs2979470	8	30288272	t	c	0.4873	0.2114	0.046	4.25E-06	Novel:one-stage design
rs2354862	8	64501744	a	c	0.6441	0.2139	0.0485	1.03E-05	Novel:one-stage design
rs13253358	8	68920135	t	c	0.297	0.1945	0.0504	0.000113	Novel:one-stage design
rs61040371	8	8503700	t	c	0.6221	0.191	0.0475	5.68E-05	Novel:one-stage design
rs62526122	8	92769569	a	g	0.2707	0.1739	0.0557	0.001806	Novel:one-stage design
rs1986971	8	10268736	a	g	0.7048	0.2632	0.051	2.49E-07	Novel:two-stage design
rs4598218	8	129483956	t	c	0.614	0.1523	0.048	0.001523	Novel:two-stage design
rs4129585	8	143312933	a	c	0.4438	0.1977	0.0467	2.30E-05	Novel:two-stage design
rs1906672	8	38130025	a	g	0.2275	0.2644	0.055	1.51E-06	Novel:two-stage design
rs6996733	8	60535824	t	c	0.8439	0.1904	0.0647	0.003269	Novel:two-stage design
rs72688070	8	81393697	t	c	0.1714	-0.1536	0.0621	0.01338	Novel:two-stage design
rs62491354	8	9730663	a	g	0.1401	0.3376	0.0663	3.59E-07	Novel:two-stage design
rs35783704	8	105966258	a	g	0.1092	-0.5219	0.0773	1.50E-11	Previously reported

rs2898290	8	11433909	t	c	0.4835	0.3419	0.0466	2.12E-13	Previously reported
rs4841569	8	11452177	a	g	0.4123	-0.3758	0.0511	1.94E-13	Previously reported
rs6557876	8	25900675	t	c	0.2511	-0.3667	0.0533	5.98E-12	Previously reported
rs520015	9	211762	c	g	0.5144	0.2043	0.0456	7.60E-06	Novel:one-stage design
rs9886665	9	22942770	t	c	0.2721	0.1887	0.0519	0.000277	Novel:one-stage design
rs60191654	9	753648	a	g	0.8143	-0.2311	0.0584	7.50E-05	Novel:one-stage design
rs7023828	9	128498594	t	c	0.423	-0.2466	0.0464	1.10E-07	Novel:two-stage design
rs1891730	9	130309028	t	c	0.6198	-0.1749	0.0479	0.000257	Novel:two-stage design
rs184457	9	131940019	a	g	0.2995	-0.1157	0.0498	0.02015	Novel:two-stage design
rs28558845	9	4334791	c	g	0.1568	-0.2472	0.0652	0.00015	Novel:two-stage design
rs1332813	9	9350706	t	c	0.3515	0.1771	0.0472	0.000175	Novel:two-stage design
rs7045409	9	95201540	a	t	0.3681	-0.1498	0.0473	0.001553	Novel:two-stage design
rs111245230	9	113169775	t	c	0.9662	-0.6917	0.1299	9.99E-08	Previously reported
rs11592107	10	122968964	a	g	0.3087	0.2721	0.0495	3.93E-08	Novel:one-stage design
rs72834453	10	124235226	t	g	0.8742	-0.2378	0.0712	0.000839	Novel:one-stage design
rs3802517	10	28233469	a	t	0.4668	0.188	0.0456	3.80E-05	Novel:one-stage design
rs11187142	10	94468685	t	c	0.1047	0.298	0.0763	9.34E-05	Novel:one-stage design
rs11197813	10	118523933	a	g	0.7025	-0.1612	0.0505	0.0014	Novel:two-stage design
rs7912283	10	133773019	a	g	0.642	-0.2008	0.0505	7.02E-05	Novel:two-stage design
rs1133400	10	134459388	a	g	0.7954	-0.307	0.0601	3.24E-07	Novel:two-stage design
rs34130368	10	48411796	t	g	0.1197	-0.1772	0.0816	0.02998	Novel:two-stage design
rs56352451	10	5804865	t	c	0.1337	0.3049	0.0672	5.69E-06	Novel:two-stage design
rs12572586	10	74751579	t	c	0.9383	-0.4496	0.1012	8.86E-06	Novel:two-stage design
rs112184198	10	102604514	a	g	0.1058	-0.5331	0.0761	2.40E-12	Previously reported
rs1004467	10	104594507	a	g	0.9028	0.8884	0.0785	1.08E-29	Previously reported
rs11191548	10	104846178	t	c	0.9129	1.0233	0.0818	6.19E-36	Previously reported
rs4746172	10	75855842	t	c	0.7348	-0.1017	0.0528	0.0542	Previously reported
rs932764	10	95895940	a	g	0.5561	-0.3654	0.0467	4.84E-15	Previously reported
rs10766533	11	19224677	a	t	0.7004	0.2572	0.0515	5.85E-07	Novel:one-stage design
rs11031051	11	30355707	a	c	0.683	-0.1902	0.0493	0.000116	Novel:two-stage design

rs190194639	11	34068037	t	c	0.0823	0.3274	0.0862	0.000146	Novel:two-stage design
rs1585453	11	46884713	a	t	0.8866	-0.2449	0.0775	0.00157	Novel:two-stage design
rs4385883	11	51539339	a	t	0.7047	0.2189	0.0566	0.000112	Novel:two-stage design
rs4980515	11	63744609	t	c	0.504	0.227	0.0464	1.01E-06	Novel:two-stage design
rs67976715	11	68023742	c	g	0.2282	0.2708	0.0555	1.04E-06	Novel:two-stage design
rs10743086	11	8774923	a	g	0.2086	-0.2193	0.0567	0.000111	Novel:two-stage design
rs7129220	11	10350538	a	g	0.1233	0.3919	0.0724	6.28E-08	Previously reported
rs1401454	11	16250183	t	c	0.3998	0.3365	0.0469	7.10E-13	Previously reported
rs757081	11	17351683	c	g	0.6644	-0.2958	0.0487	1.29E-09	Previously reported
rs5219	11	17409572	t	c	0.3755	0.32	0.0471	1.12E-11	Previously reported
rs661348	11	1905292	t	c	0.5632	-0.3417	0.0502	9.56E-12	Previously reported
rs217727	11	2016908	a	g	0.192	0.3626	0.061	2.85E-09	Previously reported
rs11537751	11	47587452	t	c	0.0521	0.3936	0.1076	0.000256	Previously reported
rs11229457	11	58207203	t	c	0.2144	-0.2886	0.0563	2.97E-07	Previously reported
rs3741378	11	65408937	t	c	0.1328	-0.4169	0.0696	2.15E-09	Previously reported
rs7927515	11	76125330	a	c	0.3455	0.1705	0.0488	0.000479	Previously reported
rs117206641	12	133086888	t	c	0.1145	0.3348	0.0783	1.88E-05	Novel:one-stage design
rs28621435	12	13860990	a	g	0.1187	-0.3138	0.0729	1.69E-05	Novel:one-stage design
rs4143175	12	67782397	t	c	0.239	0.3055	0.0533	9.90E-09	Novel:one-stage design
rs5742643	12	102837863	t	c	0.2505	-0.2603	0.0534	1.07E-06	Novel:two-stage design
rs11112548	12	105871914	a	t	0.9558	0.5768	0.1203	1.64E-06	Novel:two-stage design
rs11571376	12	1059556	c	g	0.7011	-0.1164	0.0506	0.0215	Novel:two-stage design
rs2024385	12	12888438	a	t	0.4186	-0.243	0.0467	1.99E-07	Novel:two-stage design
rs7976167	12	24210599	t	c	0.6893	0.1409	0.0489	0.003922	Novel:two-stage design
rs10437954	12	58003922	a	g	0.9064	-0.4326	0.0832	2.01E-07	Novel:two-stage design
rs7963801	12	79685226	t	c	0.4129	-0.2145	0.0482	8.45E-06	Novel:two-stage design
rs10858966	12	90567026	c	g	0.3035	0.2024	0.05	5.12E-05	Novel:two-stage design
rs2384550	12	115352731	a	g	0.3457	-0.2748	0.0473	6.29E-09	Previously reported
rs1126930	12	49399132	c	g	0.0343	0.5757	0.14	3.93E-05	Previously reported
rs73099903	12	53440779	t	c	0.0794	0.4218	0.0878	1.56E-06	Previously reported

rs7297416	12	54443090	a	c	0.6867	0.2816	0.05	1.84E-08	Previously reported
rs2681492	12	90013089	t	c	0.8344	0.7729	0.0615	3.26E-36	Previously reported
rs17249754	12	90060586	a	g	0.1637	-0.8015	0.0619	2.16E-38	Previously reported
rs2480171	13	21559858	t	c	0.1324	0.2057	0.0693	0.002978	Novel:one-stage design
rs1331012	13	27115424	t	g	0.269	0.1514	0.051	0.002962	Novel:one-stage design
rs4274337	13	41967193	a	g	0.177	-0.33	0.0612	6.93E-08	Novel:one-stage design
rs75961402	13	56398286	a	g	0.1516	0.2759	0.0635	1.40E-05	Novel:one-stage design
rs606950	13	22298923	a	g	0.6176	0.1755	0.047	0.000186	Novel:two-stage design
rs9532243	13	32191408	a	c	0.4797	0.2485	0.0452	3.89E-08	Novel:two-stage design
rs73187288	13	42738672	a	c	0.8935	-0.2492	0.0738	0.000731	Novel:two-stage design
rs912434	13	47189928	t	g	0.7628	0.2107	0.0531	7.30E-05	Novel:two-stage design
rs9526707	13	51489186	a	g	0.3166	-0.2364	0.0492	1.56E-06	Novel:two-stage design
rs78474310	13	73826901	a	g	0.9566	-0.4412	0.1138	0.000106	Novel:two-stage design
rs7988232	13	79808655	a	g	0.4146	0.1378	0.0463	0.002917	Novel:two-stage design
rs3011549	13	113634937	a	c	0.2888	0.226	0.0539	2.78E-05	Previously reported
rs63418562	13	30146201	t	c	0.7462	-0.3846	0.0529	3.74E-13	Previously reported
rs34983854	14	39858442	a	g	0.6064	-0.2259	0.0463	1.05E-06	Novel:one-stage design
rs8014182	14	103859962	t	c	0.1388	-0.3218	0.0655	8.80E-07	Novel:two-stage design
rs17115145	14	30122409	t	c	0.3909	0.1853	0.0462	6.08E-05	Novel:two-stage design
rs72683923	14	50735947	t	c	0.9767	0.7823	0.1705	4.45E-06	Novel:two-stage design
rs11623535	14	72462381	a	g	0.7393	0.1623	0.0513	0.001552	Novel:two-stage design
rs11159091	14	75074316	a	g	0.4654	0.1973	0.046	1.78E-05	Novel:two-stage design
rs9888615	14	53377540	t	c	0.2936	-0.2356	0.0499	2.32E-06	Previously reported
rs8016306	14	63928546	a	g	0.7931	0.1339	0.0554	0.01569	Previously reported
rs4965529	15	100145224	a	c	0.1657	-0.2802	0.0622	6.60E-06	Novel:two-stage design
rs11634028	15	76276150	a	t	0.205	0.2356	0.059	6.49E-05	Novel:two-stage design
rs3743157	15	85680532	a	c	0.1651	0.2069	0.0615	0.000766	Novel:two-stage design
rs11632436	15	86295286	c	g	0.5045	0.1907	0.0458	3.07E-05	Novel:two-stage design
rs35199222	15	81013037	a	g	0.4398	0.2436	0.0466	1.75E-07	Previously reported
rs2759308	15	81016227	a	g	0.4758	0.2592	0.046	1.79E-08	Previously reported

rs2379829	16	3538873	c	g	0.728	-0.2143	0.0521	3.84E-05	Novel:one-stage design
rs34941092	16	50550137	a	g	0.1491	-0.302	0.0651	3.53E-06	Novel:one-stage design
rs1012089	16	74171973	c	g	0.4758	-0.1354	0.0456	0.002974	Novel:one-stage design
rs3851018	16	86437811	c	g	0.5676	0.2224	0.0473	2.60E-06	Novel:one-stage design
rs6540125	16	87993889	t	g	0.3501	0.1864	0.0475	8.75E-05	Novel:one-stage design
rs35450617	16	6889675	t	g	0.6958	-0.1542	0.051	0.002489	Novel:two-stage design
rs7187540	16	85318302	a	c	0.3245	-0.193	0.0563	0.000606	Novel:two-stage design
rs9899540	17	30777924	a	t	0.4126	0.1809	0.0487	0.0002	Novel:one-stage design
rs112260610	17	64252393	t	c	0.1353	0.3389	0.0669	4.11E-07	Novel:one-stage design
rs4925159	17	18185510	a	g	0.4192	0.2134	0.0464	4.23E-06	Novel:two-stage design
rs1551355	17	30032420	t	c	0.2369	0.1842	0.0538	0.000621	Novel:two-stage design
rs34430710	17	56876627	a	t	0.6753	-0.2151	0.0487	9.87E-06	Novel:two-stage design
rs1036902	17	58950791	t	c	0.8404	-0.2107	0.0634	0.000888	Novel:two-stage design
rs112280096	17	79367409	a	c	0.37	-0.0932	0.0561	0.09643	Novel:two-stage design
rs12946454	17	43208121	a	t	0.739	-0.3193	0.0518	7.30E-10	Previously reported
rs7406910	17	46688256	t	c	0.0893	-0.4877	0.0812	1.93E-09	Previously reported
rs8068318	17	59483766	t	c	0.7271	0.4318	0.0536	8.20E-16	Previously reported
rs2240736	17	59485393	t	c	0.7328	0.4265	0.0525	4.49E-16	Previously reported
rs1154214	18	24546824	t	g	0.3963	-0.2163	0.046	2.57E-06	Novel:one-stage design
rs6567160	18	57829135	t	c	0.7644	0.1618	0.0541	0.002765	Novel:one-stage design
rs10460108	18	73034151	a	g	0.4819	0.2039	0.0452	6.40E-06	Novel:one-stage design
rs11876341	18	48799991	a	g	0.6949	-0.2167	0.0518	2.89E-05	Novel:two-stage design
rs10048404	18	54578482	t	c	0.3741	-0.2123	0.049	1.46E-05	Novel:two-stage design
rs12454712	18	60845884	t	c	0.6224	0.1891	0.0537	0.000429	Novel:two-stage design
rs34413141	18	777282	a	t	0.1796	-0.337	0.0599	1.83E-08	Novel:two-stage design
rs12958173	18	42141977	a	c	0.3	0.3518	0.0495	1.21E-12	Previously reported
rs7256564	19	33889593	a	g	0.3133	0.2039	0.0487	2.87E-05	Novel:one-stage design
rs73046792	19	49605705	a	g	0.1513	-0.2413	0.069	0.000474	Novel:one-stage design
rs2613765	19	5066330	a	g	0.4768	-0.1874	0.0455	3.85E-05	Novel:two-stage design
rs138877676	19	50935809	t	g	0.0211	-0.5482	0.2033	0.006999	Novel:two-stage design

rs17638167	19	11584818	t	c	0.047	-0.5228	0.1095	1.81E-06	Previously reported
rs8105753	19	31927547	a	c	0.6255	0.1895	0.0487	9.88E-05	Previously reported
rs4247374	19	7252756	t	c	0.1355	-0.5063	0.0753	1.76E-11	Previously reported
rs1764975	20	4101290	a	t	0.7894	0.2759	0.058	1.99E-06	Novel:one-stage design
rs6021247	20	50108980	a	g	0.5289	0.1623	0.0453	0.000338	Novel:two-stage design
rs6031435	20	42797358	a	g	0.5388	-0.2268	0.0456	6.72E-07	Previously reported
rs11701033	21	33788341	c	g	0.8169	-0.2465	0.0592	3.18E-05	Previously reported
rs9608690	22	28921347	a	g	0.0678	-0.308	0.0912	0.000733	Novel:one-stage design
rs28578714	22	50727921	t	c	0.6045	0.2346	0.0538	1.28E-05	Novel:two-stage design

* Candidate SNPs selected from Evangelou et al)[1]

† Regression coefficient and corresponding standard error derived from International Consortium for Blood Pressure Genome-Wide Association Studies (ICBP). [4]

Table S5. Post-hoc sensitivity analysis of the effect of systolic blood pressure lowering per se on the risk of new-onset type 2 diabetes, stratified by different diabetes ascertainment methods reported by each trial.

Trial name	Trial Design	Diabetes ascertainment type as outcome	Diabetes ascertainment type at baseline	Treatment	Comparator	Treatment		Comparator		BP difference	Incidence rate	HR (95% CIs)
						Events	Total	Events	Total			
ACTIVE I	Placebo	AE	History/glucose lowering treatment	ARBs	Placebo	196	3614	213	3617	2.91	13.6	
ALLHAT	Drug-drug	FPG	History/FPG	Diuretics	ACEIs, CCBs and ARBs	1374	9719	1810	17393	1.45	24.3	
ANBP	Placebo	AE	History/diagnosis	Diuretics	Placebo	14	1717	13	1704	9.74	2.2	
ANBP2	Drug-drug	AE	History/diagnosis	Diuretics	ACEIs	184	2817	127	2795	1.85	13.6	
ASCOT-BPLA	Drug-drug	FPG	FPG/GTT/ glucose lowering treatment/history	CCBs	BBs	565	7032	792	6982	3.52	18.3	
CAPPP	Drug-drug	FPG	FPG/GTT	BBs/Diuretics	ACEIs	380	5205	337	5154	1.18	12.0	
CASEJ	Drug-drug	ICD-self report	FPG/HbA1c/GTT/ glucose lowering treatment	CCBs	ARBs	59	1293	38	1302	2.5	11.9	
COLM	Drug-drug	AE	History/FPG/GTT	ARBs and Diuretics	ARBs and CCBs	15	1840	11	1844	0.01	2.3	
COPE	Drug-drug	AE	History/diagnosis	CCBs and ARBs	CCBs and Diuretics or CCBs and BBs	20	956	69	1871	0.36	8.7	
HIJCREATE	Drug-drug	FPG	FPG/ glucose lowering treatment	ARBs	No- ARBs	7	645	18	624	0.09	4.9	
MOSES	Drug-drug	AE	History/diagnosis	CCBs	ARBs	11	416	19	433	3.24	10.7	
NORDIL	Drug-drug	AE	History/diagnosis	BBs/Diuretics	CCBs	249	5026	216	4980	3.26	10.9	
ONTARGET	Drug-drug	FPG	History/diagnosis	ACEIs/ARBs	ARBs and ACEIs	323	5280	761	10717	2.52	14.8	

PEACE	Placebo	AE	History/diagnosis	ACEIs	Placebo	334	3417	399	3457	5.04	22.4	
PROGRESS	Placebo	AE	History/diagnosis	ACEIs/Diuretics	Placebo	80	2657	86	2685	8.35	8.0	
STOP2	Drug-drug	FPG	History/diagnosis	BBs/Diuretics	ACEIs or CCBs	97	1954	190	3923	2.57	10.8	
SYSTEUR	Placebo	ICD-self report	History/diagnosis/FPG	CCBs	Placebo	107	2165	78	2069	9.46	16.8	
TRANSCEND	Placebo	FPG	History/diagnosis/FPG	ARBs	Placebo	205	1889	238	1905	5	25.0	
PRoFESS	Placebo	AE	History/diagnosis	ARBs	Placebo	112	7108	136	7103	4.41	6.9	

**Diagnosis subgroups**		
Overall estimation (n trials=19)	15.7	0.89 (0.84 to 0.95)
Subgroup 1: Both outcome and baseline diabetes ascertained using at least one laboratory test (n trials=5) *	19.8	0.64 (0.56 to 0.73)
Subgroup 2: Outcome ascertained using at least one laboratory test (n trials=7) †	18.3	0.63 (0.55 to 0.72)
Subgroup 3: Outcome reported as AE (n trials =10) ‡	11.3	0.92 (0.84 to 1.00)
Subgroup 4: Outcome ascertained using at least one laboratory test or ICD codes-self report (n trials =9) §	18.1	0.87 (0.79 to 0.95)

Drug-drug: drug-drug comparison trials; Placebo: Placebo-controlled trial; ICD: International Classification of Diseases code; AE: adverse event, GTT: Glucose Tolerance Test; FPG: fasting plasma glucose test; History: history of type 2 diabetes; diagnosis: diagnosis of type 2 diabetes by clinical staff; BP difference: systolic blood pressure difference; Incidence rate: overall incidence rate per 1000 person-years of follow-up, CCBs: calcium channel-blockers; ACEIs: angiotensin-converting enzyme inhibitors; ARBs: angiotensin II receptor blockers; BBs: beta blockers; HR and 95% CI: hazard ratio and 95% confidence intervals standardized for 5 mmHg reduction in systolic blood pressure

* Included trials: ALLHAT, ASCOT-BPLA, CAPPP, HIJCREATE, TRANSCEND

† Included trials: ALLHAT, ASCOT-BPLA, CAPPP, HIJCREATE, TRANSCEND, ONTARGET, STOP2

‡ Included trials: ACTIVE I, ANBP, ANBP2, COLM, COPE, MOSES, NORDIL, PEACE, PROGRESS, PRoFESS

§ Included trials: ALLHAT, ASCOT-BPLA, CAPPP, HIJCREATE, TRANSCEND, ONTARGET, STOP2, SYSTEUR, CASEJ

Table S6. Post-hoc one-stage Cox proportional hazards model included random effects terms and adjusted for multiple potential confounders.

Model number	Sensitivity analysis	HR (95% CI)
#1	**Main model (fixed effect)**	0.89 (0.84 to 0.95)
	**Main model with different adjustment levels for baseline variables**	
#2	Adjusted for age and sex	0.89 (0.84 to 0.95)
#3	Adjusted for variables in model #2 + SBP at baseline	0.89 (0.84 to 0.95)
#4	Adjusted for variables in model #3 + BMI	0.90 (0.84 to 0.96)
#5	Adjusted for variables in model #4 + Comorbidities	0.88 (0.82 to 0.95)
#6	Adjusted for variables in model #5 + previous use of non-study antihypertensive medications	0.88 (0.81 to 0.94)
#7	Adjusted for variables in model #6 + previous use of non-study medications (anti-platelet drug, anticoagulants, lipid-lowering treatment)	0.86 (0.75 to 1.00)
	**Post-hoc sensitivity analysis model (random effect)**	
#8	Age as random effect term	0.91 (0.86 to 0.97)
#9	Sex as random effect term	0.91 (0.86 to 0.97)
#10	SBP categories at baseline as random effect term	0.91 (0.86 to 0.97)
#11	BMI categories as random effect term	0.92 (0.86 to 0.98)
#12	Comorbidities as random effect term	0.88 (0.83 to 0.94)
#13	Previous use of non-study antihypertensive medications as random effect term	0.93 (0.86 to 0.99)
#14	Previous use of non-study medications (anti-platelet drug, anticoagulants, lipid-lowering treatment) as random effect term	0.88 (0.80 to 0.97)

SBP: systolic blood pressure, HR: hazard ratio, CI: confidence intervals

Table S7. Structure of data used for Bayesian network meta-analysis.

Trial	Treatment	Log odds ratio	Standard error
ACTIVE I	ARBs	-0.087282	0.1019051
ACTIVE I	Placebo	Ref	Ref
ALLHAT	ACEIs	Ref	0.042723
ALLHAT	Alpha-blockers	-0.236321	0.0635346
ALLHAT	CCBs	0.180828	0.0586936
ALLHAT	Diuretics	0.34054	0.0516992
ANBP	Diuretics	0.065873	0.3867451
ANBP	Placebo	Ref	Ref
ANBP2	Diuretics	0.290631	0.1127574
ANBP2	ACEIs	Ref	Ref
ASCOT-BPLA	BBs	Ref	Ref
ASCOT-BPLA	CCBs	-0.379775	0.0578355
CAPPP	BBs and diuretics	0.119263	0.0775375
CAPPP	ACEIs	Ref	Ref
CASEJ	ARBs	Ref	Ref
CASEJ	CCBs	0.456953	0.2117247
COLM	Diuretics	0.306425	0.3983511
COLM	CCBs	Ref	Ref
COPE	ARBs	Ref	0.2260231
COPE	BBs	0.657745	0.2814929
COPE	Diuretics	0.503684	0.2888821
HOPE	ACEIs	-0.421015	0.1303484
HOPE	Placebo	Ref	Ref
INSIGHT	Diuretics	0.272714	0.1178478
INSIGHT	CCBs	Ref	Ref
MOSES	ARBs	Ref	Ref
MOSES	CCBs	-0.455182	0.359962
NORDIL	BBs and diuretics	0.141555	0.095181
NORDIL	CCBs	Ref	Ref
ONTARGET	ACEIs	Ref	0.0541296
ONTARGET	ARBs or ACEIs	-0.104219	0.0789173
ONTARGET	ARBs	0.119676	0.0751049
PEACE	ACEIs	-0.18624	0.0784146
PEACE	Placebo	Ref	Ref
PROGRESS	ACEIs and/or Diuretics	-0.062958	0.1568992
PROGRESS	Placebo	Ref	Ref
STOP2	ACEIs	Ref	0.1036344
STOP2	BBs and/or Diuretics	-0.005437	0.1469285
STOP2	CCBs	-0.052368	0.1484213
SYSTEUR	CCBs	0.28115	0.1521744
SYSTEUR	Placebo	Ref	Ref
TRANSCEND	ARBs	-0.16907	0.1003129
TRANSCEND	Placebo	Ref	Ref
VALUE	ARBs	Ref	Ref
VALUE	CCBs	0.256895	0.0555286
PRoFESS	ARBs	-0.19831	0.1287212
PRoFESS	Placebo	Ref	Ref

Ref: reference category for calculation of odds ratio
Analysis comparing the effects by drug classes were not standardized for the intensity of blood pressure reduction. This was to account for potential variations in blood pressure-lowering efficacy, tolerability, or non-blood pressure-mediated effects of the different drug classes.
CCBs: calcium channel-blockers; ACEIs: angiotensin-converting enzyme inhibitors; ARBs: angiotensin II receptor blockers; BBs: beta-blockers
ACTIVE I: Atrial Fibrillation Clopidogrel Trial with Irbesartan for Prevention of Vascular Events; ALLHAT: Antihypertensive and Lipid Lowering Treatment to Prevent Heart Attack Trial; ANBP: Australian National Blood Pressure Study; ANBP2: Second Australian National Blood Pressure Study; ASCOT-BPLA: Anglo-Scandinavian Cardiac Outcomes Trial-Blood Pressure Lowering Arm; CAPPP: Captopril Prevention Project; CASE-J: Candesartan Antihypertensive Survival Evaluation in Japan Trial; COLM: Combination of OLMesartan study; COPE: Combination Therapy of Hypertension to Prevent Cardiovascular Events; HIJ-CREATE: Heart Institute of Japan Candesartan Randomized Trial for Evaluation in Coronary Artery Disease; HOPE: Heart Outcomes Prevention Evaluation; INSIGHT: International Nifedipine GITS study: Intervention as a Goal in Hypertension Treatment; MOSES: Morbidity and Mortality After Stroke, Eprosartan Compared With Nitrendipine for Secondary Prevention; NORDIL: Nordic Diltiazem Study; ONTARGET: Ongoing Telmisartan Alone and in Combination with Ramipril Global Endpoint Trial; PEACE: Prevention of Events with Angiotensin-Converting Enzyme Inhibition; PRoFESS: Prevention Regimen for Effectively Avoiding Second Strokes; PROGRESS: Perindopril Protection Against Recurrent Stroke Study; STOP Hypertension-2: Swedish Trial in Old Patients with Hypertension-2; Syst-Eur: Systolic Hypertension in Europe; TRANSCEND: Telmisartan Randomized Assessment Study in ACE Intolerant Subjects with Cardiovascular Disease; VALUE: Valsartan Antihypertensive Long-term Use Evaluation

Supplementary Figures

Figure S1. Trial selection flowchart.

Adapted from the flowchart of the main protocol [76]

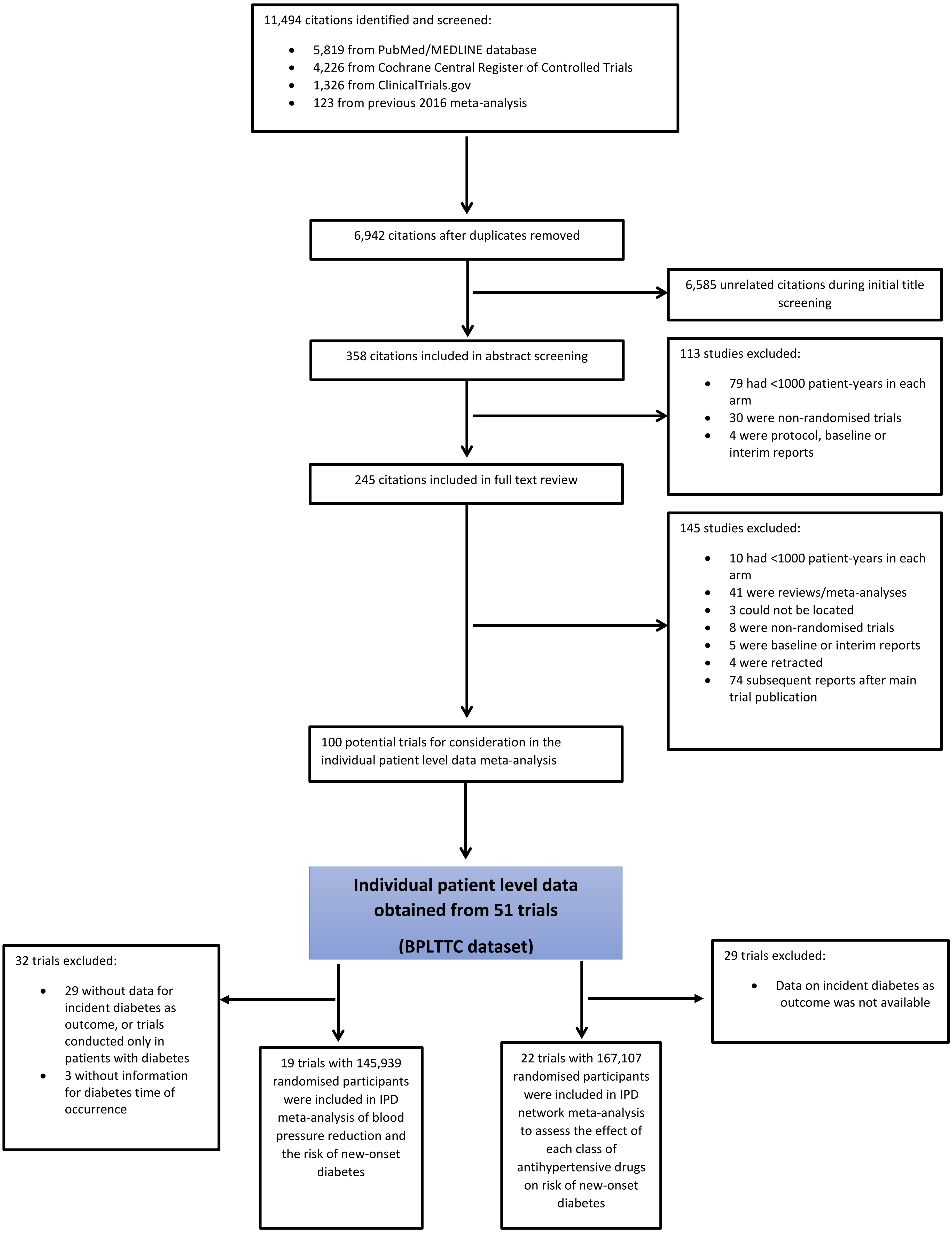

Figure S2. The genetic variant selection workflow for systolic blood pressure.
LD: linkage disequilibrium; GWAS: genome-wide association study

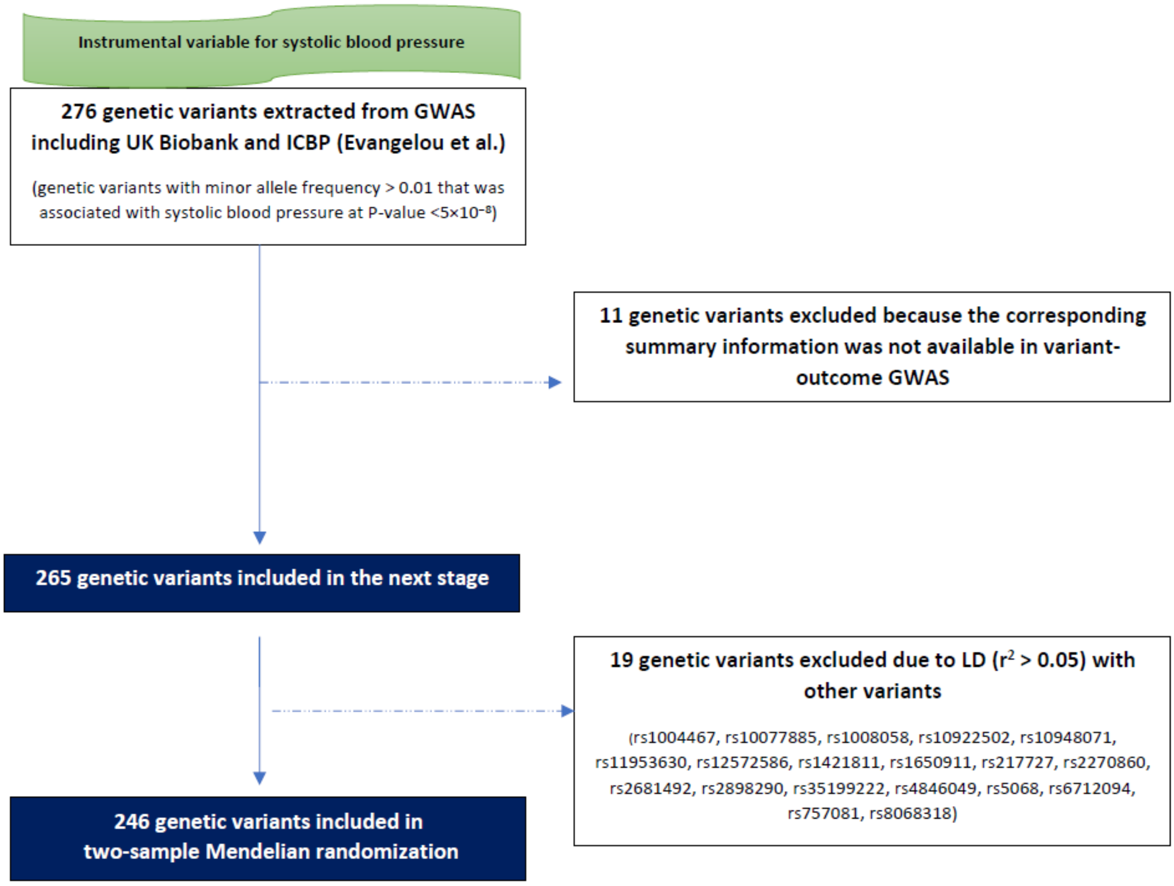

Figure S3. Forest plot showing the effect of systolic blood pressure lowering per se on the risk of new-onset type 2 diabetes, overall and separately for each trial.

The estimated heterogeneity indexes were I^2 = 86% and tau^2 = 0.11. The hazard ratio (HR) is standardised for blood pressure reduction between included trials. BP difference: systolic blood pressure difference, ACEI: angiotensin converting enzyme inhibitors, ARB: angiotensin II receptor blockers, BB: beta-blockers, CI: confidence intervals, ICD: international classification of diseases diagnosis codes

			Treatment		Comparator					
**Trials**	**Treatment**	**Comparator**	**Events**	**Total**	**Events**	**Total**	**BP diff**	**HR**	**95% CI**	**Weight**
ACTIVE I	ARB	Placebo	196	3614	213	3617	2.91	0.86	[0.62; 1.20]	3.4%
ALLHAT	Diuretic	ACEI, CCB and ARB	1374	9719	1810	17393	1.45	1.99	[1.56; 2.54]	6.4%
ANBP	Diuretic	Placebo	14	1717	13	1704	9.74	1.00	[0.68; 1.48]	2.5%
ANBP2	Diuretic	ACEI	184	2817	127	2795	1.85	2.78	[1.51; 5.11]	1.0%
ASCOT-BPLA	CCB	BB	565	7032	792	6982	3.52	0.58	[0.50; 0.68]	15.9%
CAPPP	BB/Diuretic	ACEI	380	5205	337	5154	1.18	1.64	[0.88; 3.06]	1.0%
CASEJ	CCB	ARB	59	1293	38	1302	2.50	2.43	[1.07; 5.49]	0.6%
COLM	ARB and Diuretic	ARB and CCB	15	1840	11	1844	0.01			0.0%
COPE	CCB and ARB	CCB and Diuretic or CCB and BB	20	956	69	1871	0.36			0.0%
HIJCREATE	ARB	No- ARB	7	645	18	624	0.09			0.0%
MOSES	CCB	ARB	11	416	19	433	3.24	0.47	[0.15; 1.48]	0.3%
NORDIL	BB/Diuretic	CCB	249	5026	216	4980	3.26	1.21	[0.92; 1.60]	4.8%
ONTARGET	ACEI/ARB	ARB and ACEI	323	5280	761	10717	2.52	0.75	[0.58; 0.97]	5.6%
PEACE	ACEI	Placebo	334	3417	399	3457	5.04	0.83	[0.72; 0.96]	18.0%
PROGRESS	ACE/Diuretic	Placebo	80	2657	86	2685	8.35	0.96	[0.80; 1.16]	11.3%
STOP2	BB/Diuretic	ACEI or CCB	97	1954	190	3923	2.57	1.04	[0.64; 1.67]	1.7%
SYSTEUR	CCB	Placebo	107	2165	78	2069	9.46	1.15	[0.99; 1.34]	15.7%
TRANSCEND	ARB	Placebo	205	1889	238	1905	5.00	0.86	[0.71; 1.03]	10.7%
PRoFESS	ARB	Placebo	112	7108	136	7103	4.41	0.80	[0.45; 1.40]	1.2%
**Overall**								**0.89**	**[0.84; 0.95]**	

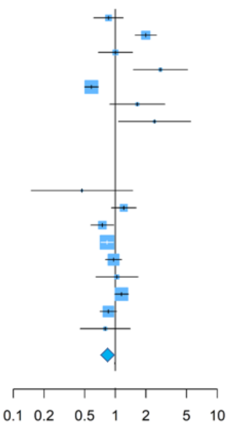

Figure S4. Funnel plot for assessment of publication (acquisition) bias on the effect of blood pressure reduction and risk of new-onset type 2 diabetes.

Egger's regression test: T statistics = - 0.2, df = 17, bias coefficient -0.22, standard error 1.09, p-value = 0.83.

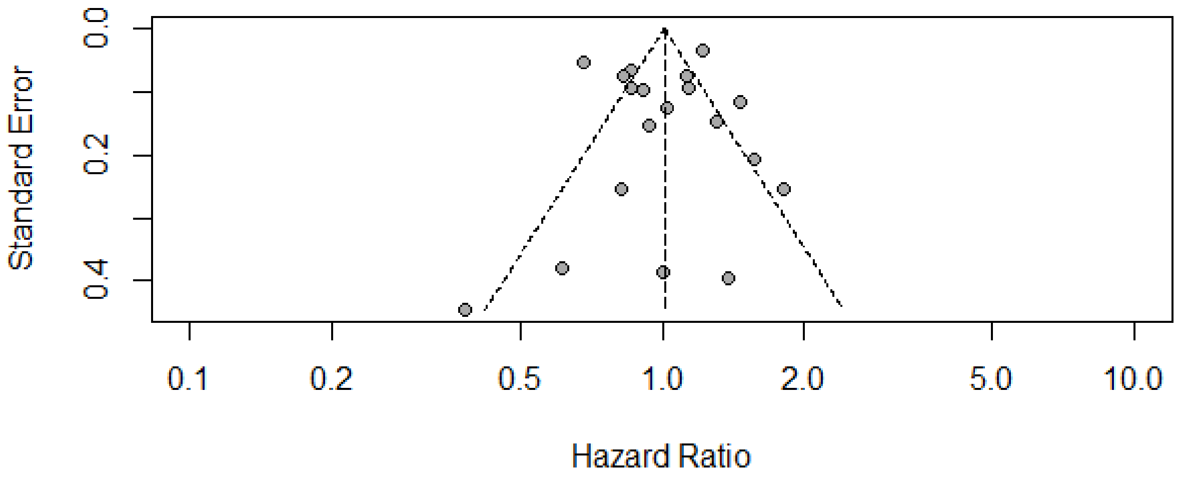

Figure S5. Absolute risk reduction for the effect of each major antihypertensive drug class on the risk of new-onset type 2 diabetes.

Absolute risk reduction (ARR) was estimated using a Poisson regression model with an identity link. The unit is absolute risk difference between each drug class versus placebo (or treatment versus comparator groups for overall effect). ARR for overall estimation was -0.0022 (95% CI -0.0049 to 0.0005). BP: systolic blood pressure, ACEIs: angiotensin-converting enzyme inhibitors, ARBs: angiotensin II receptor blockers, CI: confidence intervals.

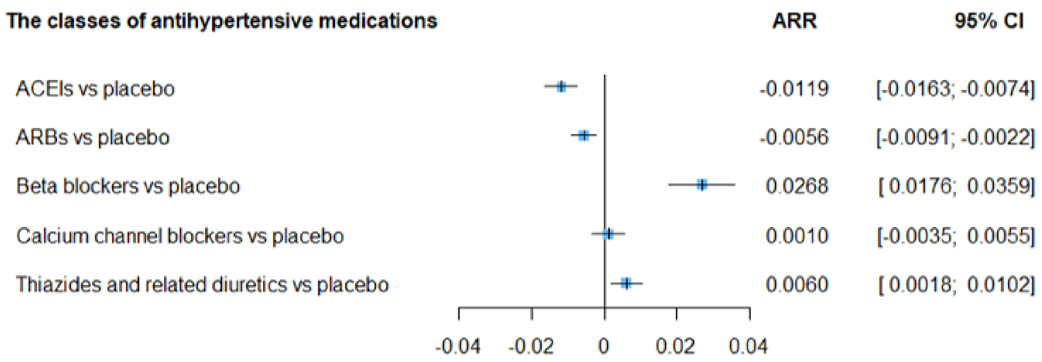

Figure S6. Effect of systolic blood pressure lowering per se on the risk of new-onset type 2 diabete.

In IPD meta-analysis, the effect size was standardised for blood pressure reduction between included trials.

Blue squares represent the point estimation and size of squares is the same. The horizontal solid lines represent 95% confidence intervals. Relative risk: indicates hazard ratio in IPD meta-analysis of randomised controlled trials and odds ratio in Mendelian randomisation, IPD: individual participant data, RCT: randomised controlled trial, CI: confidence interval.

Study	No. of events	Total sample size	Relative risk per 5 mmHg reduction in systolic blood pressure (95% CI)
IPD meta-analysis of RCTs	9883	135425	0.89 [0.84; 0.95]
Mendelian randomization	21147	455607	0.88 [0.84; 0.92]

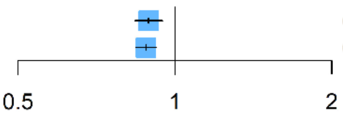

Each 5 mmHg genetically-influenced lower systolic blood pressure was associated with a 12% lower risk of type 2 diabetes (OR: 0.88 [95% CI 0.84 to 0.92]). The positive control analysis showed strong associations for the same magnitude of systolic blood pressure difference and risk of coronary heart disease (OR: 0.87 [95% CI 0.84 to 0.90]), myocardial infarction (OR: 0.90 [95% CI 0.87 to 0.93]), and ischemic stroke (OR: 0.85 [95% CI 0.80 to 0.91]), further supporting the validity of our instrumental variable (**Figure S7**). The Mendelian randomisation regression slopes are shown in **Figures S8** and **S9.** Overall, estimated ORs based on different methods were similar in magnitude and direction, suggesting that there was no material effect of pleiotropy on the causal estimation (**Figure S10**). In the leave-one-out analysis, we found that no single genetic variant was noticeably driving the overall effect of systolic blood pressure on type 2 diabetes (**Figure 11**). Consistent results from the sensitivity analyses also support our main findings (**Figures S12** -**S14**).

Figure S7. Mendelian randomisation estimates for the association between genetically predicted 5-mmHg systolic blood pressure reduction and diabetes as the main outcome, and coronary heart disease, myocardial infarction, and ischemic stroke as positive control outcomes.

Blue squares represent the point estimation and size of squares is the same. The horizontal solid lines represent 95% confidence intervals. Cases and controls: number of cases and controls in genome-wide association studies. Odds ratio (OR): estimated using the inverse-variance weighted method

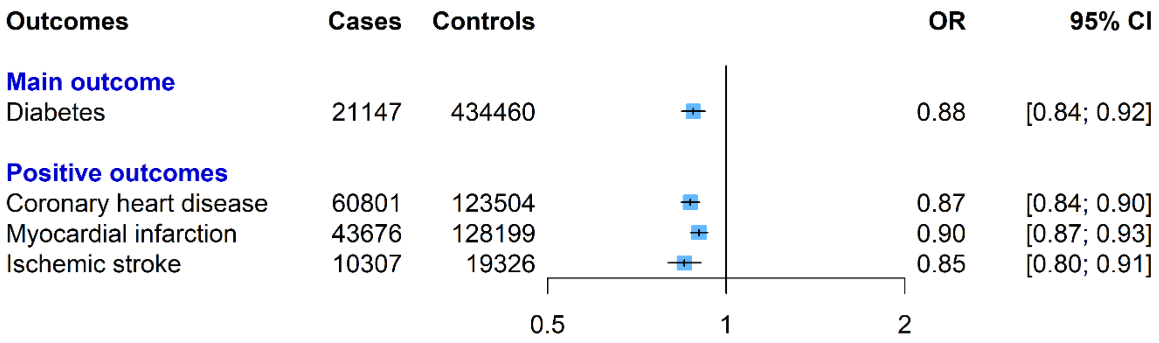

Figure S8. Scatter plot of genetic variant-outcome associations versus variant-exposure associations for the association between systolic blood pressure and risk of type 2 diabetes.

Circles indicate marginal genetic associations with systolic blood pressure and risk of diabetes for each variant. Error bars indicate 95% CIs.
SNP: single-nucleotide polymorphism

Panel A (Scatter plot including outlier variants) **Panel B** (Scatter plot excluding outlier variants)

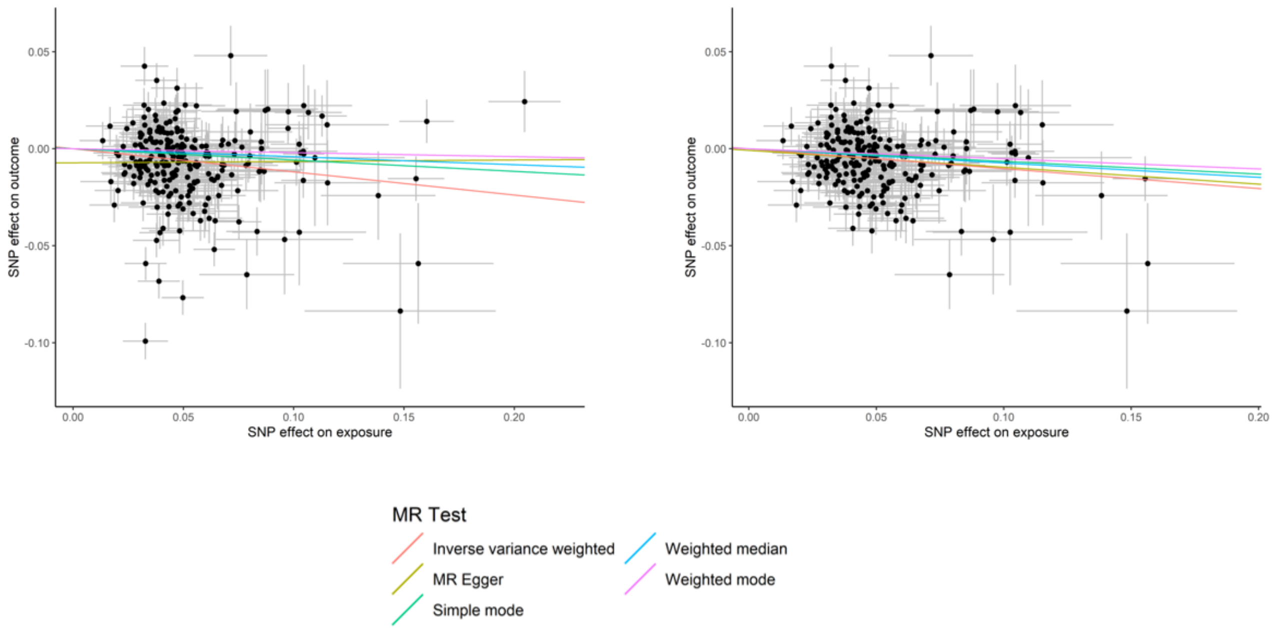

Figure S9. Funnel plot of variants, showing each variant causal estimate against instrument strength.

β: The causal effect of the exposure on the outcome, SE: standard error

Panel A (Funnel plot including outlier variants) **Panel B** (Funnel plot excluding outlier variants)

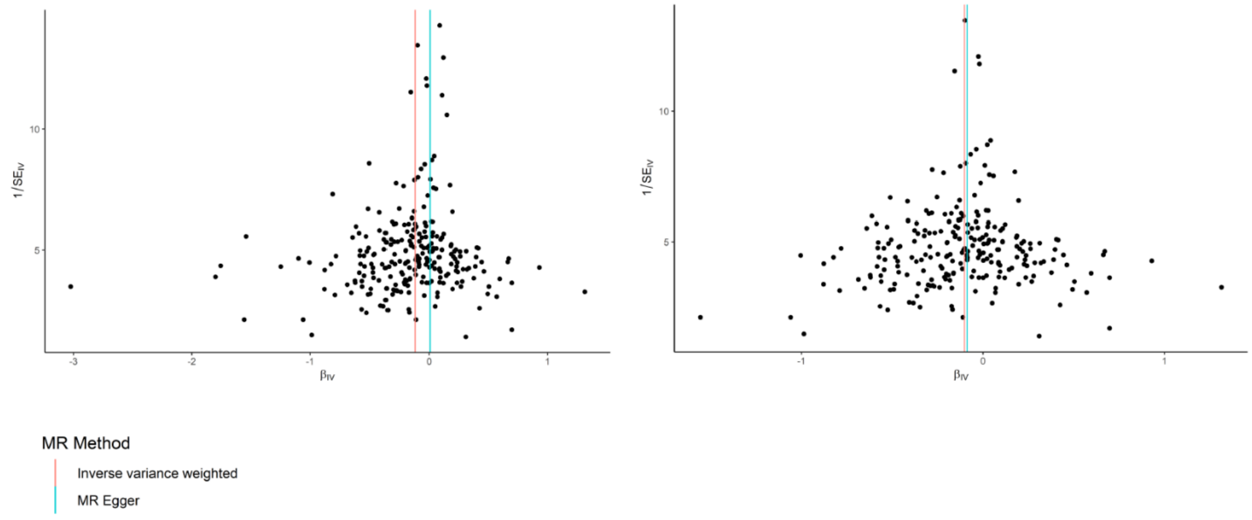

Figure S10. The association between systolic blood pressure and risk of diabetes estimated by random-effect inverse variance weighted and applied various sensitivity analysis methods of two-sample Mendelian randomisation, before (main analysis) and after excluding outlier variants (sensitivity analysis).

Blue squares represent the point estimation and size of squares is the same. The horizontal solid lines represent 95% confidence intervals. The MBE method was implemented using both simple and weighted options with bandwidth φ = 1 under the no measurement error (NOME) assumption. The following outlier variants excluded based on Cook's distance measure over 4/n, where n is the number of included genetic variants: rs10274928, rs11191548, rs12454712, rs1446468, rs17249754, rs17477177, rs2972146, rs34072724, rs4841569, rs5219, rs6712203, rs9368222

Panel A (Analyses including outlier variants)

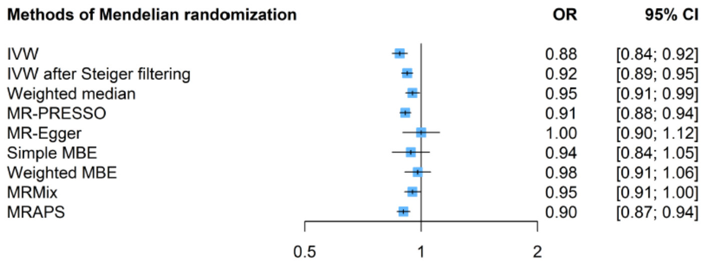

Panel B (Analyses excluding outlier variants)

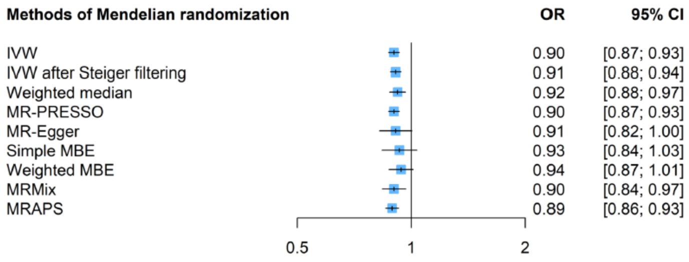

Figure S11. Leave-one-out plot to assess if a single variant is driving the association between systolic blood pressure and diabetes.

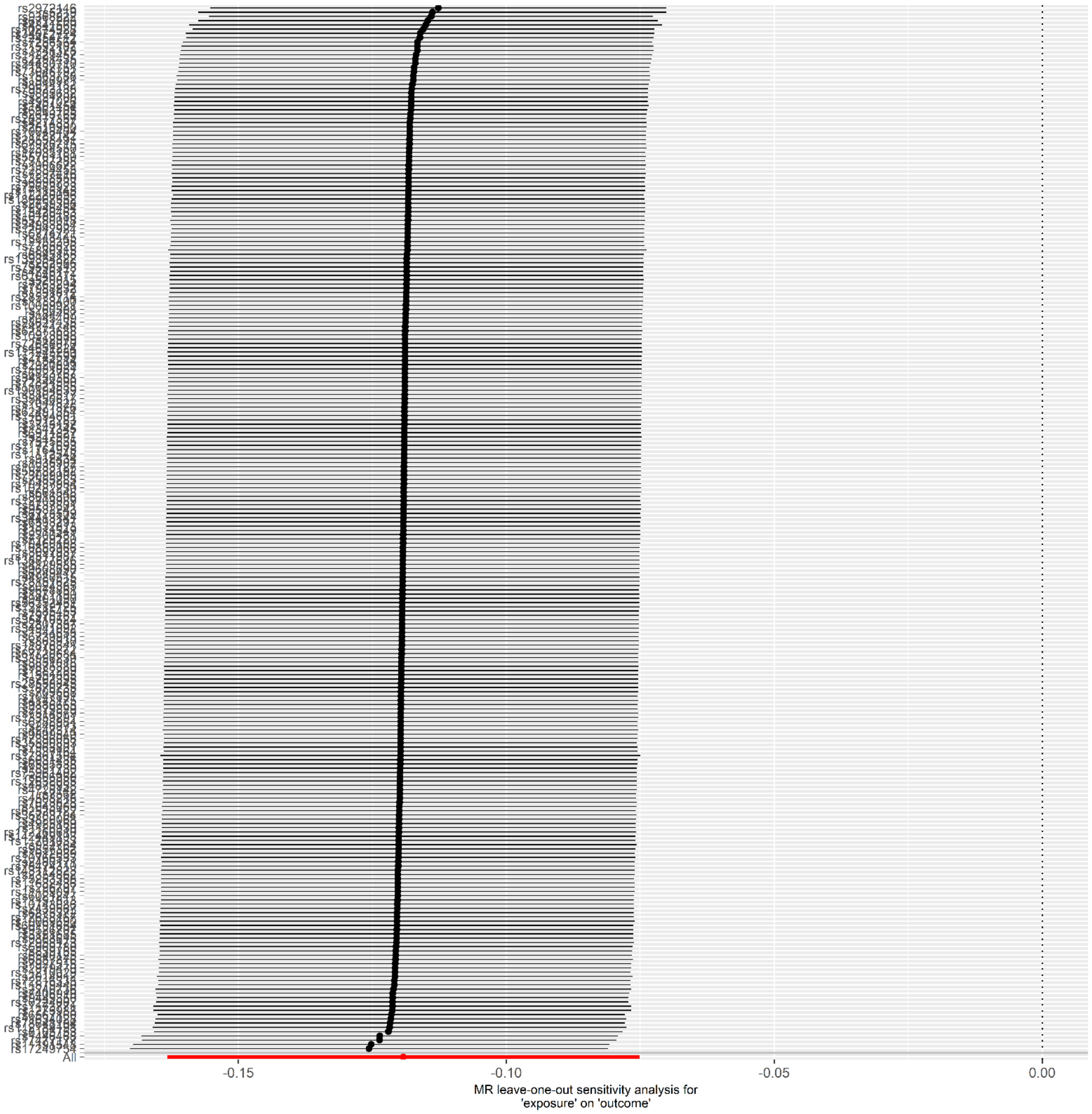

Figure S12. Multivariable Mendelian randomisation results unadjusted and adjusted for the anthropometric measures to check the possibility of collider bias in association between systolic blood pressure and diabetes.

Blue squares represent the point estimation and size of squares is the same. The horizontal solid lines represent 95% confidence intervals. In conventional Mendelian randomisation analysis, we used the summary statistics not adjusted for body mass index or other anthropometric measures. Multivariable Mendelian randomisation adjusted for body mass index, waist circumference, hip circumference, and fat percentage; OR: odds ratio per 5 mmHg lower systolic blood pressure, IVW: inverse-variance weighted

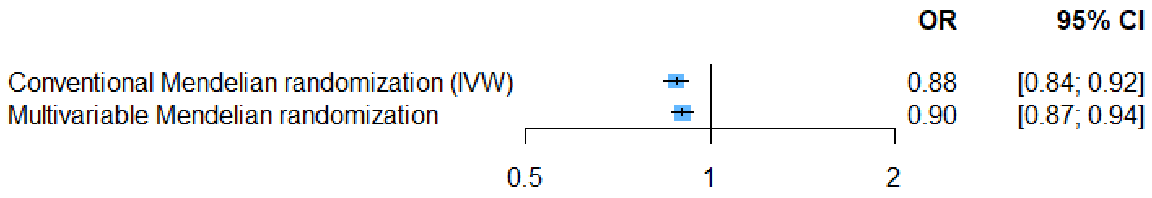

Figure S13. Sensitivity analysis to assess the impact of using overlap dataset for the discovery of candidate instrumental variable for exposure.

The below forest plot shows the association between systolic blood pressure and risk of diabetes estimated by random-effect inverse variance weighted and applied various sensitivity analyses. The instrumental variable (IV)-exposure estimations were the same as the main analysis, but IV-outcome summary estimations were extracted from stage 1 of the DIAbetes Genetics Replication And Meta-analysis (DIAGRAM) Consortium, which has no overlap with the UK Biobank. Blue squares represent the point estimation and size of squares is the same. The horizontal solid lines represent 95% confidence intervals. OR: odds ratio per 5- mmHg lower systolic blood pressure

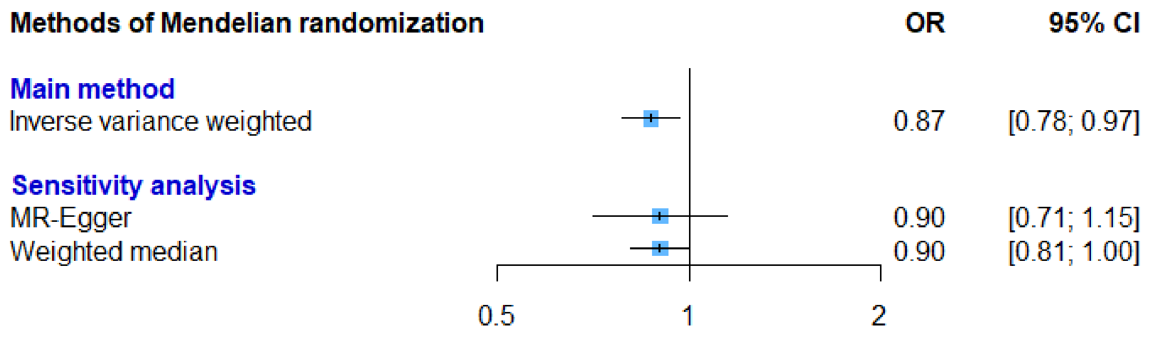

Figure S14. The association between systolic blood pressure and risk of diabetes replicated by one-sample Mendelian randomisation.

The result of the main one-sample analysis, in which genetic variants were the same as in the two-sample Mendelian randomisation, was similar to the main findings (OR 0.87 [95% CI 0.84 to 0.90]). In addition to this, we have conducted a new one-sample Mendelian randomisation. In this sensitivity analysis, to build a new genetic risk score, we selected 370 genetic variants from the final International Consortium for Blood Pressure (ICBP) GWAS dataset included 77 cohorts (n = 299,024, no overlap with UK Biobank). The result of this sensitivity analysis using a new built genetic risk score was in line with the previous one-sample analysis (OR 0.88 [95% CI 0.85 to 0.92]).

Blue squares represent the point estimation and size of squares is the same. The horizontal solid lines represent 95% confidence intervals. OR: odds ratio per 5 mmHg lower systolic blood pressure

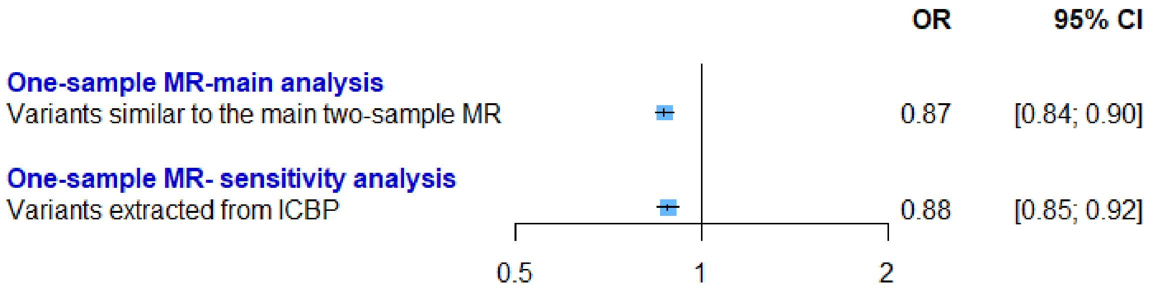

Figure S15. Association of genetically influenced systolic blood pressure reduction overall and for each major class of antihypertensive medications, with type 2 diabetes as the main outcome, and coronary heart disease as the positive control.

Blue squares represent the point estimation and size of squares is the same. The horizontal solid lines represent 95% confidence intervals. Odds ratio: estimated using the inverse-variance weighted method. Effect of blood pressure-lowering class of drugs estimated using genetic variants in genes encode drug targets. ACEIs/ARBs: angiotensin-converting enzyme inhibitors/ angiotensin receptor blockers. CI: confidence interval

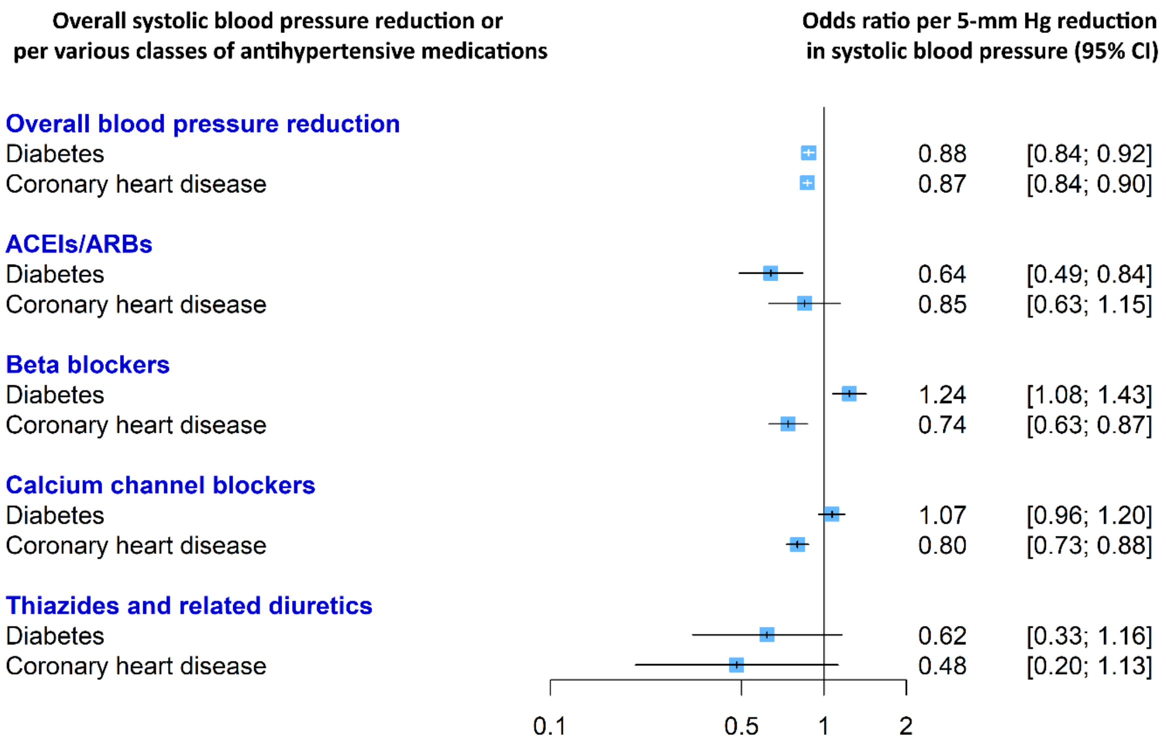

In the positive control analysis, systolic blood pressure reduction predicted through genetic variants for ACEIs/ARBs, beta-blockers and CCBs were associated with a lower risk of coronary heart disease as an established evidence-based target for preventive blood pressure-lowering treatment, supporting the validity of the gene-based analysis. For type 2 diabetes, we found a decrease in the risk with ACEIs/ARBs, null effect with CCBs and increased risk with beta-blockers. The genetic evidence for thiazide diuretics did not provide adequate statistical power for replication.

References

1 Evangelou E, Warren HR, Mosen-Ansorena D, *et al.* Genetic analysis of over 1 million people identifies 535 new loci associated with blood pressure traits. *Nature Genetics* 2018; **50**: 1412–25.

2 Xue A, Wu YY, Zhu Z, *et al.* Genome-wide association analyses identify 143 risk variants and putative regulatory mechanisms for type 2 diabetes. *Nature Communications* 2018; **9**: 2941.

3 Hartwig FP, Davies NM, Hemani G, Davey Smith G. Two-sample Mendelian randomization: avoiding the downsides of a powerful, widely applicable but potentially fallible technique. *International journal of epidemiology* 2016; **45**: 1717–26.

4 Ehret GB, Munroe PB, Rice KM, *et al.* Genetic variants in novel pathways influence blood pressure and cardiovascular disease risk. *Nature* 2011; **478**: 103–9.

5 Fortier I, Raina P, Van den Heuvel ER, *et al.* Maelstrom Research guidelines for rigorous retrospective data harmonization. *International Journal of Epidemiology* 2016; **46**: dyw075.

6 Hartwig FP, Smith GD, Bowden J. Robust inference in summary data Mendelian randomization via the zero modal pleiotropy assumption. *International Journal of Epidemiology* 2017; **46**: 1985–98.

7 Hemani G, Bowden J, Davey Smith G. Evaluating the potential role of pleiotropy in Mendelian randomization studies. Human molecular genetics. 2018; **27**: R195–208.

8 Bowden J, Davey Smith G, Haycock PC, Burgess S. Consistent Estimation in Mendelian Randomization with Some Invalid Instruments Using a Weighted Median Estimator. *Genetic epidemiology* 2016; **40**: 304–14.

9 Verbanck M, Chen C-Y, Neale B, Do R. Detection of widespread horizontal pleiotropy in causal relationships inferred from Mendelian randomization between complex traits and diseases. *Nature Genetics* 2018; **50**: 693–8.

10 Bowden J, Davey Smith G, Burgess S. Mendelian randomization with invalid instruments: effect estimation and bias detection through Egger regression. *International Journal of Epidemiology* 2015; **44**: 512–25.

11 Burgess S, Thompson SG. Interpreting findings from Mendelian randomization using the MR-Egger method. *European Journal of Epidemiology* 2017; **32**: 377–89.

12 Cook RD. Detection of Influential Observation in Linear Regression. *Technometrics* 1977; **19**: 15.

13 Zhao Q, Wang J, Hemani G, Bowden J, Small DS. Statistical inference in two-sample summary-data Mendelian randomization using robust adjusted profile score. *Annals of Statistics* 2018; **48**: 1742–69.

14 Qi G, Chatterjee N. Mendelian randomization analysis using mixture models for robust and efficient estimation of causal effects. *Nature Communications* 2019; **10**: 1–10.

15 Hemani G, Tilling K, Davey Smith G. Orienting the causal relationship between imprecisely measured traits using GWAS summary data. *PLoS Genetics* 2017; **13**. DOI:10.1371/journal.pgen.1007081.

16 Burgess S, Bowden J, Fall T, Ingelsson E, Thompson SG. Sensitivity Analyses for Robust Causal Inference from Mendelian Randomization Analyses with Multiple Genetic Variants.

Epidemiology 2017; **28**: 30–42.

17 Hemani G, Zheng J, Elsworth B, *et al.* The MR-base platform supports systematic causal inference across the human phenome. *eLife* 2018; **7**. DOI:10.7554/eLife.34408.

18 Yavorska OO, Burgess S. MendelianRandomization: An R package for performing Mendelian randomization analyses using summarized data. *International Journal of Epidemiology* 2017; **46**: 1734–9.

19 Neale B. Rapid GWAS of thousands of phenotypes in the UK Biobank 2020. 2020. http://www.nealelab.is/blog/2017/7/19/rapid-gwas-of-thousands-of-phenotypes-for-337000-samples-in-the-uk-biobank (accessed April 19, 2020).

20 Sanderson E, Davey Smith G, Windmeijer F, Bowden J. An examination of multivariable Mendelian randomization in the single-sample and two-sample summary data settings. *International Journal of Epidemiology* 2019; **48**: 713–27.

21 Burgess S, Thompson SG. Multivariable Mendelian Randomization: The Use of Pleiotropic Genetic Variants to Estimate Causal Effects. *American Journal of Epidemiology* 2015; **181**: 251–60.

22 Nikpay M, Goel A, Won HH, *et al.* A comprehensive 1000 Genomes-based genome-wide association meta-analysis of coronary artery disease. *Nature Genetics* 2015; **47**: 1121–30.

23 Malik R, Traylor M, Pulit SL, *et al.* Low-frequency and common genetic variation in ischemic stroke. *Neurology* 2016; **86**: 1217–26.

24 Morris AP, Voight BF, Teslovich TM, *et al.* Large-scale association analysis provides insights into the genetic architecture and pathophysiology of type 2 diabetes. *Nature Genetics* 2012; **44**: 981–90.

25 Sudlow C, Gallacher J, Allen N, *et al.* UK biobank: an open access resource for identifying the causes of a wide range of complex diseases of middle and old age. *PLoS medicine* 2015; **12**: e1001779.

26 UK Biobank Coordinating Centre. UK Biobank: Protocol for a large-scale prospective epidemiological resource UK Biobank Coordinating Centre. Design. 2007; **06**: 1–112.

27 Bycroft C, Freeman C, Petkova D, *et al.* The UK Biobank resource with deep phenotyping and genomic data. *Nature* 2018; **562**: 203–9.

28 Nazarzadeh M, Pinho-Gomes AC, Smith Byrne K, *et al.* Systolic Blood Pressure and Risk of Valvular Heart Disease: A Mendelian Randomization Study. *JAMA Cardiology* 2019; **4**: 788–95.

29 Lewis CJ, Gong H, Brown MJ, Harding SE. Overexpression of β 1-adrenoceptors in adult rat ventricular myocytes enhances CGP 12177A cardiostimulation: Implications for 'putative' β 4-adrenoceptor pharmacology. *British Journal of Pharmacology* 2004; **141**: 813–24.

30 Nazarzadeh M, Pinho-Gomes AC, Bidel Z, *et al.* Genetic susceptibility, elevated blood pressure, and risk of atrial fibrillation: a Mendelian randomization study. *Genome Medicine* 2021; **13**: 38.

31 Gill D, Georgakis MK, Koskeridis F, *et al.* Use of Genetic Variants Related to Antihypertensive Drugs to Inform on Efficacy and Side Effects. *Circulation* 2019; **140**: 270–9.

32 Walker VM, Kehoe PG, Martin RM, Davies NM. Repurposing antihypertensive drugs for the prevention of Alzheimer's disease: a Mendelian randomization study. *International Journal of Epidemiology* 2019; published online July 23. DOI:10.1093/ije/dyz155.

33 Law MR, Morris JK, Wald NJ. Use of blood pressure lowering drugs in the prevention of cardiovascular disease: Meta-analysis of 147 randomised trials in the context of expectations from prospective epidemiological studies. *BMJ (Online)* 2009; **338**: 1245.

34 Jamerson K, Weber MA, Bakris GL, *et al.* Benazepril plus Amlodipine or Hydrochlorothiazide for Hypertension in High-Risk Patients. *New England Journal of Medicine* 2008; **359**: 2417–28.

35 Furberg CD, Wright JT, Davis BR, *et al.* Major outcomes in high-risk hypertensive patients randomized to angiotensin-converting enzyme inhibitor or calcium channel blocker vs diuretic: The antihypertensive and lipid-lowering treatment to prevent heart attack trial (ALLHAT). *Journal of the American Medical Association* 2002; **288**: 2981–97.

36 Ettehad D, Emdin CA, Kiran A, *et al.* Blood pressure lowering for prevention of cardiovascular disease and death: a systematic review and meta-analysis. *Lancet (London, England)* 2016; **387**: 957–67.

37 Gill D, Georgakis MK, Koskeridis F, *et al.* Use of Genetic Variants Related to Antihypertensive Drugs to Inform on Efficacy and Side Effects. *Circulation* 2019; **140**: 270–9.

38 Walker VM, Kehoe PG, Martin RM, Davies NM. Repurposing antihypertensive drugs for the prevention of Alzheimer’s disease: a Mendelian randomization study. *International Journal of Epidemiology* 2019; : 1–9.

39 Emdin CA, Anderson SG, Woodward M, Rahimi K. Usual Blood Pressure and Risk of New-Onset Diabetes Evidence from 4.1 Million Adults and a Meta-Analysis of Prospective Studies. *Journal of the American College of Cardiology* 2015; **66**: 1552–62.

40 Roumie CL, Hung AM, Russell GB, *et al.* Blood Pressure control and the association with Diabetes Incidence: Results from the SPRINT Randomized trial. *Hypertention* 2019; **75**: 331–8.

41 Sun D, Zhou T, Heianza Y, *et al.* Type 2 Diabetes and Hypertension. *Circulation research* 2019; **124**: 930–7.

42 Aikens RC, Zhao W, Saleheen D, *et al.* Systolic blood pressure and risk of type 2 diabetes: A mendelian randomization study. *Diabetes* 2017; **66**: 543–50.

43 Zhu Z, Zheng Z, Zhang F, *et al.* Causal associations between risk factors and common diseases inferred from GWAS summary data. *Nature Communications* 2018; **9**: 224–224.

44 Gress TW, Nieto FJ, Shahar E, Wofford MR, Brancati FL. Hypertension and Antihypertensive Therapy as Risk Factors for Type 2 Diabetes Mellitus. *New England Journal of Medicine* 2000; **342**: 905–12.

45 Fletcher A, Amery A, Birkenh Ä Ger W, *et al.* Risks and benefits in the trial of the european working party on high blood pressure in the elderly. *Journal of Hypertension* 1991; **9**: 225–30.

46 Savage PJ, Pressel SL, Curb JD, *et al.* Influence of long-term, low-dose, diuretic-based, antihypertensive therapy on glucose, lipid, uric acid, and potassium levels in older men and women with isolated systolic hypertension: The Systolic Hypertension in the Elderly Program. *Archives of Internal Medicine* 1998; **158**: 741–51.

47 Cooper-DeHoff R, Cohen JD, Bakris GL, *et al.* Predictors of Development of Diabetes Mellitus in Patients With Coronary Artery Disease Taking Antihypertensive Medications (Findings from the INternational VErapamil SR-Trandolapril STudy [INVEST]). *American Journal of Cardiology* 2006; **98**: 890–4.

48 Elliott WJ, Meyer PM. Incident diabetes in clinical trials of antihypertensive drugs: a network meta-analysis. *Lancet* 2007; **369**: 201–7.

49 Yusuf S, Healey JS, Pogue J, *et al.* Irbesartan in patients with atrial fibrillation. *New England Journal of Medicine* 2011; **364**: 928–38.

50 Grimm RH, Margolis KL, Papademetriou V, *et al.* Baseline characteristics of participants in the Antihypertensive and Lipid Lowering Treatment to Prevent Heart Attack Trial (ALLHAT). *Hypertension* 2001; **37**: 19–27.

51 Doyle AE. The Australian National blood pressure study. *Trends in Pharmacological Sciences* 1981; **2**: 293–6.

52 Wing LMH, Reid CM, Ryan P, *et al.* A comparison of outcomes with angiotensin-converting-enzyme inhibitors and diuretics for hypertension in the elderly. *New England Journal of Medicine* 2003; **348**: 583–92.

53 Gupta AK, Dahlof B, Dobson J, Sever PS, Wedel H, Poulter NR. Determinants of new-onset diabetes among 19,257 hypertensive patients randomized in the anglo-scandinavian cardiac outcomes trial-blood pressure lowering arm and the relative influence of antihypertensive medication. *Diabetes Care* 2008; **31**: 982–8.

54 Hansson L, Lindholm LH, Niskanen L, *et al.* Effect of angiotensin-converting-enzyme inhibition compared with conventional therapy on cardiovascular morbidity and mortality in hypertension: the Captopril Prevention Project (CAPPP) randomised trial. *Lancet* 1999; **353**: 611–6.

55 Nakao K, Hirata M, Oba K, *et al.* Role of diabetes and obesity in outcomes of the candesartan antihypertensive survival evaluation in Japan (CASE-J) trial. *Hypertension Research* 2010; **33**: 600–6.

56 Ogihara T, Saruta T, Rakugi H, *et al.* Rationale, study design and implementation of the COLM study: The combination of OLMesartan and calcium channel blocker or diuretic in high-risk elderly hypertensive patients. *Hypertension Research* 2009; **32**: 163–7.

57 Matsuzaki M, Ogihara T, Umemoto S, *et al.* Prevention of cardiovascular events with calcium channel blocker-based combination therapies in patients with hypertension: A randomized controlled trial. *Journal of Hypertension* 2011; **29**: 1649–59.

58 Kasanuki H, Hagiwara N, Hosoda S, *et al.* Angiotensin II receptor blocker-based vs. non-angiotensin II receptor blocker-based therapy in patients with angiographically documented coronary artery disease and hypertension: The Heart Institute of Japan Candesartan Randomized Trial for Evaluation in. *European Heart Journal* 2009; **30**: 1203–12.

59 Sharma AM, Pischon T, Engeli S. Effect of ramipril on cardiovascular events in high-risk patients. *New England Journal of Medicine* 2000; **343**.

60 Brown MJ, Palmer CR, Castaigne A, *et al.* Morbidity and mortality in patients randomised to double-blind treatment with a long-acting calcium-channel blocker or diuretic in the International Nifedipine GITS study: Intervention as a Goal in Hypertension Treatment (INSIGHT). *Lancet* 2000; **356**: 366–72.

61 Schrader J, Lüders S, Kulschewski A, *et al.* Morbidity and mortality after stroke, eprosartan compared with nitrendipine for secondary prevention: Principal results of a prospective randomized controlled study (MOSES). *Stroke* 2005; **36**: 1218–24.

62 Hansson L, Hedner T, Lund-Johansen P, *et al.* Randomised trial of effects of calcium antagonists compared with diuretics and β-blockers on cardiovascular morbidity and mortality in hypertension: The Nordic Diltiazem (NORDIL) study. *Lancet* 2000; **356**: 359–65.

63 Mann JFE, Anderson C, Gao P, *et al.* Dual inhibition of the renin-angiotensin system in high-risk diabetes and risk for stroke and other outcomes: Results of the ONTARGET trial. *Journal of Hypertension* 2013; **31**: 414–21.

64 Braunwald E, Domanski MJ, Fowler SE, *et al.* Angiotensin-converting-enzyme inhibition in stable coronary artery disease. *New England Journal of Medicine* 2004; **351**. DOI:10.1056/NEJMoa042739.

65 Randomised trial of a perindopril-based blood-pressure-lowering regimen among 6,105 individuals with previous stroke or transient ischaemic attack. *Lancet* 2001; **358**: 1033–41.

66 Dahlof B, Hansson L, Lindholm LH, *et al.* STOP-Hypertension 2: a prospective intervention trial of ‘newer’ versus ‘older’ treatment alternatives in old patients with hypertension. Swedish Trial in Old Patients with Hypertension. *Blood Press* 1993; **2**: 136–41.

67 Gasowski J, Staessen JA, Celis H, *et al.* Systolic hypertension in Europe (Syst-Eur) trial phase 2: Objectives, protocol, and initial progress. *Journal of Human Hypertension* 1999; **13**: 135–45.

68 Telmisartan T, Assessment R. Effects of the angiotensin-receptor blocker telmisartan on cardiovascular events in high-risk patients intolerant to angiotensin-converting enzyme inhibitors: a randomised controlled trial. *The Lancet* 2008; **372**: 1174–83.

69 Julius S, Kjeldsen SE, Weber M, *et al.* Outcomes in hypertensive patients at high cardiovascular risk treated with regimens based on valsartan or amlodipine: The VALUE randomised trial. *Lancet* 2004; **363**: 2022–31.

70 Sacco RL, Diener HC, Yusuf S, *et al.* Aspirin and extended-release dipyridamole versus clopidogrel for recurrent stroke. *New England Journal of Medicine* 2008; **359**: 1238–51.

71 Dahlöf B, Sever PS, Poulter NR, *et al.* Prevention of cardiovascular events with an antihypertensive regimen of amlodipine adding perindopril as required versus atenolol adding bendroflumethiazide as required, in the Anglo-Scandinavian Cardiac Outcomes Trial-Blood Pressure Lowering Arm (ASCOT-B. *Lancet* 2005; **366**: 895–906.

72 Ogihara T, Saruta T, Rakugi H, *et al.* Combinations of olmesartan and a calciumchannel blocker or a diuretic inelderly hypertensive patients: A randomized, controlled trial. *Journal of Hypertension* 2014; **32**: 2054–63.

73 Yusuf S, Teo KK, Pogue J, *et al.* Telmisartan, ramipril, or both in patients at high risk for vascular events. *New England Journal of Medicine* 2008; **358**: 1547–59.

74 Hansson L, Lindholm LH, Ekbom T, *et al.* Randomised trial of old and new antihypertensive drugs in elderly patients: Cardiovascular mortality and morbidity the Swedish trial in old patients with hypertension-2 study. *Lancet* 1999; **354**: 1751–6.

75 Staessen J, Fagard R, Thijs L, *et al.* Randomised double-blind comparison of placebo and active treatment for older patients with isolated systolic hypertension. The Systolic Hypertension in Europe (Syst-Eur) Trial Investigators. Lancet. 1997; **350**: 757–64.

76 Rahimi K, Canoy D, Nazarzadeh M, *et al.* Investigating the stratified efficacy and safety of pharmacological blood pressure-lowering: An overall protocol for individual patient-level data meta-analyses of over 300 000 randomised participants in the new phase of the Blood Pressure Lowering Treatm. *BMJ Open* 2019; **9**. DOI:10.1136/bmjopen-2018-028698.
